# Presenting Precision Glycomacromolecules on Gold Nanoparticles for Increased Lectin Binding

**DOI:** 10.3390/polym9120716

**Published:** 2017-12-14

**Authors:** Sophia Boden, Kristina G. Wagner, Matthias Karg, Laura Hartmann

**Affiliations:** 1 Institute of Organic Chemistry and Macromolecular Chemistry, Heinrich-Heine-University Düsseldorf, Universitätsstraße 1, 40225 Düsseldorf, Germany; Sophia.Boden@hhu.de; 2 Institute of Physical Chemistry, Heinrich-Heine-University Düsseldorf, Universitätsstraße 1, 40225 Düsseldorf, Germany; Kristina.Wagner@hhu.de (K.G.W.); Matthias.Karg@hhu.de (M.K.)

**Keywords:** precision glycomacromolecules, glyco-gold nanoparticles, SPR, Con A

## Abstract

Glyco-functionalized gold nanoparticles have great potential as biosensors and as inhibitors due to their increased binding to carbohydrate-recognizing receptors such as the lectins. Here we apply previously developed solid phase polymer synthesis to obtain a series of precision glycomacromolecules that allows for straightforward variation of their chemical structure as well as functionalization of gold nanoparticles by ligand exchange. A novel building block is introduced allowing for the change of spacer building blocks within the macromolecular scaffold going from an ethylene glycol unit to an aliphatic spacer. Furthermore, the valency and overall length of the glycomacromolecule is varied. All glyco-functionalized gold nanoparticles show high degree of functionalization along with high stability in buffer solution. Therefore, a series of measurements applying UV-Vis spectroscopy, dynamic light scattering (DLS) and surface plasmon resonance (SPR) were performed studying the aggregation behavior of the glyco-functionalized gold nanoparticles in presence of model lectin Concanavalin A. While the multivalent presentation of glycomacromolecules on gold nanoparticles (AuNPs) showed a strong increase in binding compared to the free ligands, we also observed an influence of the chemical structure of the ligand such as its valency or hydrophobicity on the resulting lectin interactions. The straightforward variation of the chemical structure of the precision glycomacromolecule thus gives access to tailor-made glyco-gold nanoparticles (glyco-AuNPs) and fine-tuning of their lectin binding properties.

## 1. Introduction

As a major component of most cell surfaces, carbohydrates participate in various cell-cell interactions e.g., in cellular communication and pathogen recognition where they act as ligands for different receptors such as the lectins [1,2]. An important concept underlying carbohydrate lectin interactions is multivalency, where multiple binding epitopes of the carbohydrate ligand interact with multiple binding sites of the protein [3,4]. In this way, the overall binding strength of the otherwise weak interactions of single carbohydrate ligands substantially increases, also known as the cluster glycoside effect [5,6].

Currently, the most common strategy to study carbohydrate lectin interactions is the manifold presentation of carbohydrates on a macromolecular scaffold. For example, proteins and peptides [7,8,9,10,11], dendrimers [12], polymers [13,14,15,16], fullerenes [17,18,19], calixarenes [20,21,22,23], carbon nanotubes [24] and quantum dots [25,26,27,28,29] serve as platforms for multivalent presentation. Among these various macromolecular scaffolds, especially gold nanoparticles (AuNPs) are promising glycan displays. They are inexpensive and with their ease of synthesis and functionalization various glycoconjugates can be attached in a simple manner [30]. The obtained glyco-gold nanoparticles (glyco-AuNPs) are of comparable size to biomacromolecules providing a dense glycocalyx like surface that is composed of an extremely inert and biocompatible material [31]. Moreover, they exhibit unique optical and electronical properties [30,32,33,34]. AuNPs show an intense color in the visible region because of their localized surface plasmon resonance (LSPR). The LSPR position and strength depends strongly on the particle shape, size, dispersion media and degree of aggregation and hence represent a convenient means to monitor changes in the local environment [35,36,37]. A significant color change from red to blue can be observed upon lectin induced aggregation which led to the utilization of glyco-AuNPs as biosensors with naked eye detection [35,38,39,40,41,42,43,44,45]. Moreover, the advantageous properties of glyco-AuNPs have led to further studies on potential biological applications such as HIV vaccines [46], X-ray contrast agents [47] as well as their use for ligand screening and diagnosis [48,49].

Although it is well stated in the literature that in addition to a multivalent presentation, the ligand architecture [50], carbohydrate valency, density as well as carbohydrate type [51,52,53,54] and the spatial arrangement of carbohydrate ligands [55] can have an influence on carbohydrate lectin interactions, few studies have been performed on the effect of these factors on glyco-AuNP lectin binding. Kikkeri and coworkers found that the nanoparticle shape influences cellular uptake in different cancer cell lines [56] and studied the effect of heterogeneity of glycodendrons on bacterial binding [57]. Moreover, most studies showed an effect of the carbohydrate density and the spacer type and length in regard to undesirable aggregation in buffer medium [42,58,59] but only few studies investigated the effect of these factors on lectin binding [38,45].

**Scheme 1:**
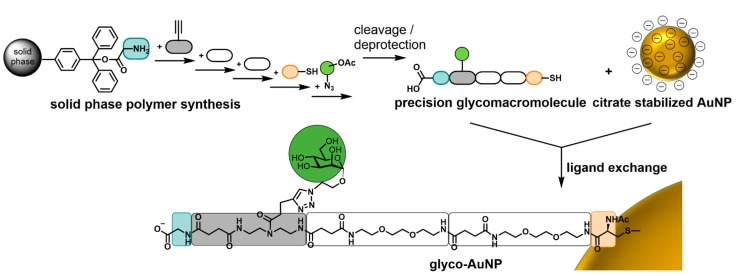
Schematic representation of the combination of solid phase polymer synthesis and nanoparticle functionalization by ligand exchange reaction.

Therefore, in this study, we want to gain first insights into the role of the structure of the carbohydrate presenting ligand attached to the AuNPs and the resulting binding behavior of glyco-AuNPs in interaction with lectins. We envision that the control over carbohydrate presentation with an adequate selection of scaffold characteristics not only leads to glyco-AuNPs with high stability but also supports the final aim of achieving high affinity and specificity for lectin binding. Thus, high control but also flexibility during the synthesis of the carbohydrate presenting ligand is required for new insights into their structure activity correlation. We fulfil this requirement by using the previously established solid phase polymer synthesis (SPPoS) [52,53,54,60,61,62] to obtain so-called precision glycomacromolecules that can then be employed for AuNP functionalization by ligand exchange reaction using a terminal thiol group introduced via a l-cysteine residue (Scheme 1). SPPoS is based on solid phase peptide synthesis [63] utilizing tailor-made building blocks and Fmoc-coupling protocols giving oligo(amidoamine) scaffolds with various functional side chains e.g., for the site specific conjugation of carbohydrate ligands. In principle, two types of building blocks are employed in SPPoS, functional building blocks that comprise different functionalities in their side chain and spacer building blocks that differ in their main chain characteristics [60,61,62,64,65]. The sequential assembly of these building blocks on solid support followed by carbohydrate conjugation gives access to monodisperse, sequence-controlled multivalent sugar mimetics, the so-called precision glycomacromolecules [54,66,67,68,69]. In dependence of the employed building blocks and their sequence during assembly, we can vary the number, position and kind of carbohydrate ligands along with the composition and architecture of the macromolecular scaffold [52,53,54,62]. Applying precision glycomacromolecules as model compounds to study multivalent effects in lectin binding, we could show a dependence of carbohydrate lectin interactions on ligand valency [52,54] and backbone as well as linker characteristics [61,62] arising from predominantly sterical, hydrophobic and statistical effects as are also known for other multivalent glycomimetics [70,71,72,73,74,75]. Here we now extend this concept by attaching a first set of precision glycomacromolecules onto AuNPs and investigate the effect of differences in the glycomacromolecular structure on the glyco-AuNP lectin binding.

First, a new aliphatic spacer building block based on 1,8-diaminooctane, ODS (octyl diamine succinic acid), is introduced based on aliphatic linkers known to form stable AuNP glycoconjugates [59] which further allows the comparison to the previously established ethylene glycol-based spacer building block EDS (ethylene glycol diamine succinic acid) [54]. Three glycomacromolecules are assembled on solid support varying the scaffold composition by using the two different spacer building blocks as well as the valency from one to five mannopyranoside ligands. Glycomacromolecules are then attached to the AuNPs and resulting glyco-AuNPs are tested for their buffer stability and degree of functionalization. In order to examine how valency and backbone properties affect the binding to model lectin receptor Concanavalin A (Con A), aggregation behavior with the lectin is tested by UV-Vis absorbance spectroscopy and the inhibitory potency of the glyco-AuNPs is assessed in an inhibition-competition assay in SPR. 

## 2. Materials and Methods

Diethyl ether (with BHT as inhibitor, ≥99.8%), triisopropylsilane (TIPS) (98%), (+)-sodium-l-ascorbate (≥99%), acetic acid (99.8%), triethylsilane (99%), conc. hydrochloric acid (p.a.), Amberlite^®^ IR 120 resin (hydrogen form), toluidine blue O (TBO), Bovine serum albumin (BSA) (≥96%), α-d-mannose (99%), sodium citrate dihydrate (≥99%), gold(III)chloride trihydrate (≥99%), *N*-ethylmaleimide (≥98%) and manganese chloride tetrahydrate (≥99%) were all purchased from Sigma-Aldrich (Steinheim, Germany). *N,N*-Diisopropylethylamine (DIPEA) (≥99%) was purchased from Carl Roth (Karlsruhe, Germany). Methanol (p.a.), concentrated sulphuric acid (95%), sodium hydroxide (1 M) and acetic anhydride (99.7%) were purchased from VWR BDH Prolabo Chemicals (Fontenay-sous-Bois, France). *N,N*-dimethylformamide (DMF) (99.8%, for peptide synthesis), piperidine (99%), 1,8-diaminooctane (98%), triphenylmethyl chloride (Trt-Cl) (98%), succinic anhydride (99%), methyl-α-d-mannopyranoside (αMeMan) (99+%) and copper(II) sulfate (98%) were purchased from Acros Organics (Geel, Belgium). Dichloromethane (DCM) (99.99%), sodium chloride (99.98%), tetrahydrofuran (THF) (analytical reagent grade), ethyl acetate (analytical reagent grade), sodium hydrogen carbonate (analytical reagent grade), toluene (analytical reagent grade), nitric acid (65%) and 4-(2-hydroxyethyl)-1-piperazineethanesulfonic acid (HEPES) (99%) were purchased from Fisher Scientific (Loughborough, UK; Fair Lawn, NJ, USA). Con A was purchased from LKT Laboratories (Biomol, Hamburg, Germany). Calcium chloride (≥97%), phenol (crystalline for analysis), potassium carbonate (for analysis), citric acid (for analysis), triethylamine (pure) and d-(+)-galactose (pure) were purchased from AppliChem (Darmstadt, Germany). Trifluoroacetic acid (TFA) (99%) and Tris(2-carboxyethyl)phosphine hydrochloride (TCEP) were purchased from Fluorochem (Hadfield, UK). 9-Fluorenylmethoxycarbonyl chloride (Fmoc-Cl) (98%) was purchased from ChemPur (Karsruhe, Germany). Sodium sulfate (anhydrous) was purchased from Caelo (Hilden, Germany). Boron trifluoride diethyl etherate (98%) and sodium methoxide (98%) were purchased from Alfa Aesar (Kandel, Germany). Sodium hydroxide (0.2 M solution), sodium chloride solution (5.0 M) and HBS-P+ buffer (0.1 M Hepes, 1.5 M NaCl, 0.5% *v/v* surfactant P20, pH 7.4) were purchased from GE Healthcare (Uppsala, Sweden). α-d-Galactose-PAA-biotin and α-d-mannose-PAA-biotin were purchased from Glyco Tech (Gaithersburg, MD, USA). Fmoc-Gly-Trt Tentagel resin (0.22 mmol/g), H-Gly-2-chlorotrityl PS resin (0.44 mmol/g) and *N*-alpha-(9-Fluorenylmethyloxycarbonyl)-*S*-trityl-l-cysteine (Fmoc-l-Cys(Trt)-OH) (99.9%) were purchased from Iris Biotech (Marktredwitz, Germany). Water was purified with a Milli-Q system (Millipore) obtaining a final resistivity of 18 MΩcm.

### 2.1. Nuclear Magnetic Resonance Spectroscopy (NMR)

^1^H-NMR and ^13^C-NMR spectroscopy were performed on a Bruker AVANCE III 300 (300 MHz) and 600 (600 MHz) (Bremen, Germany) at room temperature. Chemical shifts are referenced relative to the residual ^1^H or ^13^C peaks of the used solvent as internal standards (CDCl_3_: ^1^H 7.26, ^13^C: 77.16, DMSO-*d*_6_: ^1^H 2.50, ^13^C: 39.52, D_2_O: ^1^H 4.79). Chemical shifts are reported in delta (δ) expressed in parts per million (ppm). Multiplicities are abbreviated as follows: s = singlet, d = doublet, t = triplet, q = quartet and m = multiplet.

### 2.2. Mass Spectrometry (ESI, MALDI-TOF-MS, HR-MS)

Electrospray ionization (ESI) mass spectra were recorded with a mass spectrometer type Ion-Trap-API Finningan LCQ Deca (Thermo Quest, Austin, TX, USA).

Matrix-assisted laser desorption-ionization time-of-flight (MALDI-TOF) measurements were performed on a MALDI-TOF Ultraflex I (Bruker Daltonics, Bremen, Germany) with 2,5-dihydroxybenzoic acid (DHB) and α-cyano-4-hydroxycinnamic acid (CHCA) as matrix. The ratio of compound to matrix was 10:1.

High resolution mass spectrometry was performed on a Bruker UHR-QTOF maXis 4G instrument (Bruker Daltonics, Bremen, Germany) with a direct inlet via syringe pump, an ESI source and a quadrupole followed by a time of flight (Q-TOF) mass analyzer.

### 2.3. Reversed Phase High Pressure Liquid Chromatography—Mass Spectrometry (RP-HPLC-MS)

RP-HPLC-MS analysis was performed on an Agilent 1260 Infinity instrument (Agilent Technologies, Waldbronn, Germany) coupled to a variable wavelength detector (VWD) (set to 214 nm) and a 6120 Agilent Quadrupole MS containing an electrospray ionisation (ESI) source operating in positive ionization mode in a *m*/*z* range of 200 to 2000. As HPLC column a Poroshell 120 EC-C18 1.8 µm (3.0 × 50 mm, 2.5 µm) RP column from Agilent was used. The mobile phases A and B were 95/5 H_2_O/MeCN, 0.1% formic acid (A) and 5/95 H_2_O/MeCN, 0.1% formic acid (B). Samples were analyzed at a flow rate of 0.4 mL/min using a linear gradient from 100% A to 50% B in a time range of 30 min at 25 °C. UV and MS spectral analysis were performed within the OpenLab ChemStation software for LC/MS from Agilent Technologies (Waldbronn, Germany).

### 2.4. Preparative Reversed Phase High Pressure Liquid Chromatography (Prep-RP-HPLC)

Prep-RP-HPLC analysis was performed on an Agilent Technologies 1260 Infinity instrument (Agilent Technologies, Waldbronn, Germany) coupled to a multiple wavelength detector (MWD) (set to 214 nm). As HPLC column a CAPCELL PAK C18 (20 mm l.D. × 250 mm, 5 µM) RP column from Shiseido was used. The mobile phases A and B were H_2_O, 0.1% formic acid (A) and MeCN, 0.1% formic acid (B). Samples were purified at a flow rate of 20 mL/min at room temperature using a linear gradient from 25% B to 35% B in a time range of 10 min for compound **1**; 20% B to 26% B in 10 min for compound **3**; 20% B to 80% B in 10 min for compound **2**. The eluent fractions with the desired product were collected by an automated fraction collector and were then combined, concentrated and lyophilized.

### 2.5. UV-Vis Spectroscopy

UV-Vis absorbance measurements were performed using a dual-trace spectrometer Specord^®^ 210 Plus from Analytik Jena AG (Jena, Germany). The instrument was operated using Win Aspect Plus software (Analytik Jena AG, Jena, Germany). For Con A concentration determination, a Spectral Scan from 270–290 nm was used. The concentration of Con A expressed in terms of the monomer (*M*_w_ = 26,500 g/mol) was then determined at 280 nm applying the Beer-Lambert law with ε_280_ = 30,150 M^−1^·cm^−1^ for tetravalent ConA. For glyco-AuNP concentration determination, a Spectral Scan from 390–410 nm was used. Based on the assumption that the nanoparticles are spheres with a radius of 7 nm and that an absorbance of A = 0.3 at a wavelength of 400 nm corresponds to a gold concentration of *c*(Au^0^) = 1.25 × 10^−4^ M, the concentration of the nanoparticles was determined using the equation described by X. Liu et al. [76]. All solutions were measured in a 3.5 mL precision quartz glass cuvette (*d* = 1 cm) from Hellma Analytics (Müllheim, Germany).

### 2.6. Microtiter Plate Spectrophotometer

UV-Vis spectroscopic measurements in 96-well microplate-format were performed on a Thermo Scientific^TM^ Multiscan^TM^ GO spectrophotometer (Thermo Scientific, Vantaa, Finland) at 22 °C. The measurement was either performed in a wavelength range from 400 to 750 nm in 1 to 10 nm steps or in multiple wavelength mode measuring at 400 and 700 nm. For all experiments Corning^®^ 96-well clear flat bottom polystyrene not treated microplates (Sigma Aldrich, Steinheim, Germany) were used.

### 2.7. Freeze Dryer

Lyophilization of final glycomacromolecules was performed on an Alpha 1–4 LD plus instrument from Martin Christ Freeze Dryers GmbH (Osterode, Germany). The main drying method was set to −54 °C and 0.1 mbar.

### 2.8. Dynamic Light Scattering

Dynamic light scattering experiments were conducted at a standard goniometer setup (LS Instruments, 2D-DLS, Fribourg, Switzerland) using a HeNe-laser (632.8 nm) with a maximum constant output power of 35 mW as light source. The measurements were performed at a scattering angle of 90° and at a constant temperature of 20 °C which was achieved by a heat-controlled decalin bath connected to a circulating water bath (Julabo CF31). The light scattered by the sample was detected by two avalanche photodetectors operating in pseudo-cross-correlation mode. The resulting intensity-time autocorrelation functions were analyzed by second-order Cumulant analysis as provided by the instrument software to obtain the hydrodynamic radius. For all measurements dust-free disposable culture tubes made of borosilicate glass (Fisher Scientific, Schwerte, Germany) were used. All measurements were performed three times using acquisition times of 30 s.

### 2.9. Transmission Electron Microscopy

Transmission electron microscopy (TEM) was performed with a Zeiss E902 transmission electron microscope with an accelerating voltage of 80,000 V in bright-field mode. The sample was prepared by drop-casting of the dispersion on a carbon-coated copper grid (200 mesh, Electron Microscopy Sciences, Hatfield, MA, USA).

### 2.10. Phenol-Sulphuric Acid Method

The phenol-sulphuric acid method used for degree of functionalization determination of the glyco-AuNPs followed the procedures described previously [77,78,79]. The standard curve was generated using α-d-mannose in concentrations of 20, 40, 80, 160 and 320 µM in Milli-Q water. For detachment of the α-d-mannopyranoside moieties from the glyco-AuNPs, 200 µL of the respective nanoparticle solution were treated with 40 µL of concentrated hydrochloric acid. The mixture was stored at 80 °C for 24 h. After HCl treatment, the solutions were centrifuged (Wisespin^®^ CF-10, Witeg Labortechnik, Wertheim, Germany, 90 min, 13,500 rpm) and 100 µL of the supernatant were treated with 100 µL of 5 wt % phenol in MilliQ water and 500 µL concentrated sulphuric acid. After vigorous mixing of the vessels they were incubated at room temperature for 30 min. Then sugar concentration was determined at 490 nm in a spectral scan from 450 to 550 nm at 25 °C using the dual-trace spectrometer Specord^®^ 210 Plus from Analytik Jena AG (Jena, Germany). Milli-Q water was used as a reference. The absorption at 490 nm of a control series of the zero sample (100 µL citrate stabilized nanoparticles after HCl treatment, 100 µL 5 wt % aqueous phenol solution, 500 µL concentrated sulphuric acid) were subtracted from absorption at 490 nm. All measurements were performed in triplicates. Through the comparison to the standard curve, glycomacromolecule concentration was calculated and compared with nanoparticle concentration resulting in the degree of functionalization. All solutions were placed in a disposable polystyrene cuvette (10 × 4 × 45 mm) from Sarstedt (Nümbrecht, Germany).

### 2.11. TBO Titration

The colorimetric titration with the dye toluidine blue O (TBO) followed a procedure adapted from a previously published general method for quantification of carboxylic end groups [80]. For TBO titration, an aqueous solution with pH 10.4 was prepared. In addition, a previously prepared 12.5 mM TBO solution (prepared at least one day in advance and stored in the dark) was diluted by factor 40. The glyco-AuNP solutions were concentrated by centrifugation (Allegra^TM^ 25 R Centrifuge, Beckman Coulter, Brea, CA, USA, 6 h, 15,000 rpm) and the supernatant was removed to give 300 µL of glyco-AuNP solutions in the concentration range of 129 to 154 nM. The concentrated solution was then distributed by transferring 50 µL of the glyco-AuNP solution into three 1.5 mL Eppendorf tubes. At the same time, three blank Eppendorf tubes were prepared that contain 50 µL of MilliQ water. To each of these six Eppendorf tubes, 283 µL of the diluted TBO solution were added. The Eppendorf tubes were covered with aluminum foil and were shaken overnight. The next day, all Eppendorf tubes were centrifuged (Beckman Coulter Allegra^TM^ 25 R Centrifuge, Beckman Coulter, Brea, CA, USA, 6 h, 15,000 rpm). Then, 100 µL of the supernatant were diluted with 567 µL of the alkaline aqueous solution. The solutions were placed into a 600 µL Hellma Analytics quartz glass cuvette and UV absorbance of the diluted solution was measured at 633 nm. By comparing blank and glyco-AuNP absorbance intensity, the concentration of carboxylic groups and thus the degree of functionalization of glyco-AuNP was determined.

### 2.12. Stability of Glyco-AuNPs in Dependence of Sodium Chloride Concentration

The stability of glyco-AuNPs in dependence of sodium chloride concentration was studied following the procedure described in literature with some modifications [58]. The colorimetric assay was performed by dividing a 10 nM solution of the glyco-AuNP solution in MilliQ water into 96-well microtiter plates (100 µL/well). 100 µL of sodium chloride solutions in MilliQ water were added to each well to obtain final concentrations of 5 nM glyco-AuNPs and serial dilutions of 0, 12.5, 25, 37.5, 50, 100, 125, 250, 500 and 1000 mM sodium chloride. After 24 h the absorption spectrum (wavelength range 400 to 750 nm, 10 nm step) was recorded. 

### 2.13. Stability of Glyco-AuNPs in Lectin Binding Buffer (LBB)

The stability of glyco-AuNPs in lectin binding buffer (LBB, pH 7.40 ± 0.01, 10 mM Hepes, 50 mM sodium chloride, 1 mM calcium chloride, 1 mM manganese chloride) was investigated similarly to sodium chloride stability. A 5 nM solution of the glyco-AuNPs in lectin binding buffer (LBB) was prepared by concentrating an aqueous solution of the glyco-AuNPs by centrifugation (Allegra^TM^ 25 R Centrifuge, Beckman Coulter, Brea, CA, USA, overnight, 25 °C, 4500 rcf) and resuspension in LBB. The 5 nM solution was divided into 96-well microtiter plates (200 µL/well). After 24 h the absorption spectrum (wavelength range 400 to 750 nm, 10 nm step) was recorded. 

### 2.14. Plate-Based Colorimetric Aggregation Study with Con A

The colorimetric aggregation study with Con A followed a procedure adapted from a previously published method for lectin induced aggregation studies by absorbance [43]. A 10 nM solution of the glyco-AuNP in lectin binding buffer (LBB) was prepared by concentrating an aqueous solution of the glyco-AuNP by centrifugation (Allegra^TM^ 25 R Centrifuge, Beckman Coulter, Brea, CA, USA, overnight, 25 °C, 4500 rcf) and resuspension in LBB. The 10 nM solution was divided into 96-well microtiter plates (100 µL/well). 100 µL of Con A serial dilutions in LBB were added to each well to obtain final concentrations of 5 nM glyco-AuNP and 0, 20, 40, 80, 100, 150, 200, 300, 500, 1000, 2000, 3500 and 10,000 nM of Con A. After 30 min the absorption spectrum (wavelength range 400 to 750 nm, 1 nm step) was recorded. Then 50 µL of αMeMan in LBB were added to give a concentration of 60 mM αMeMan in each well. After 5 min the absorption spectrum (wavelength range 400 to 750 nm, 1 nm step) was recorded. 

To test for unspecific protein adsorption, the same procedure was repeated for BSA. The 10 nM glyco-AuNP solution was divided into 96-well microtiter plates (100 µL/well). 100 µL of BSA serial dilutions in LBB were added to each well to obtain final concentrations of 5 nM glyco-AuNP and 0, 20, 200, 2000, and 10,000 nM of BSA. After 30 min the absorption spectrum (wavelength range 400 to 750 nm, 1 nm step) was recorded.

### 2.15. Kinetic Study of Aggregation Behavior with Con A by DLS and UV-Vis Spectroscopy

For the kinetic study of aggregation behavior with Con A performed by DLS and UV-Vis spectroscopy, a similar procedure as described by the group of Gibson was performed [41,42]. For both studies a 10 nM solution of glyco-AuNPs in LBB was mixed in a 1:1 ratio with Con A to obtain K_D_ concentration of Con A (209 nM for **4**, 203 nM for **5**, 357 nM for **6**) and 5 nM glyco-AuNP concentration.

For the kinetic studies using absorbance spectroscopy (Thermo Scientific, Vantaa, Finland), the stock solutions were mixed in wells of a 96-well microtiter-plate (100 µL of each solution per well). The absorbance at 400 and 700 nm was measured every minute for 30 min.

For the kinetic studies using DLS, the stock solutions were filtrated twice (Roth, Rotilabo^®^ syringe filter, Karlsruhe, Germany, PTFE, 5.0 µm) and were pre-tempered to 20 °C. Then 750 µL of each stock solution were filled into the culture tubes and mixed. DLS measurements were performed at 20 °C and at a scattering angle of 90°. Every minute an intensity-time autocorrelation function was recorded with an acquisition time of 30 s during a period of 30 min.

### 2.16. Surface Plasmon Resonance (SPR)

SPR measurements were performed on a Biacore X100 instrument from GE Healthcare Life Sciences (Uppsala, Sweden). Sensorgrams were recorded with the Biacore X100 Control Software and evaluated with the Biacore X100 Evaluation Software.

As sensor chip a SA (streptavidin) chip from GE Healthcare Life Sciences was used. The positive (α-d-Mannose-PAA-biotin, flow cell 2) and negative (α-d-Galactose-PAA-biotin, flow cell 1) control for Con A binding were immobilized on the sensor chip surface using the standard biotin streptavidin capture method as adapted from previously established protocols [54,62]. Therefore, a 4.2 µg/mL solution of both polymers was prepared in HBS-P+ buffer (pH 7.4, GE Healthcare, Uppsala, Sweden). The same buffer was used as running buffer in a flow rate of 10 µL/min. For immobilization, the immobilization wizard was used with a specified contact time of 420 s. Sodium chloride, sodium hydroxide and isopropanol solutions were prepared as specified by the manufacturer. For flow cell 1 an immobilization level of 1168.5 RU (resonance units) and for flow cell 2 an immobilization level of 842.8 RU was obtained. After approximately 500 measurement cycles, a new SA (streptavidin) chip was used for further measurements. For immobilization, the same procedure was used as described above. For flow cell 1 an immobilization level of 1153.4 RU and for flow cell 2 an immobilization level of 794.4 RU was obtained. In order to make all experiments comparable, αMeMan was used as a reference inhibitor on both SA sensor chips before and after the measurement series.

SPR inhibition competition studies were performed using the wizard template kinetics/affinity with contact times of 105 s and dissociation times of 180 s. Before the run, the system was primed with LBB (pH 7.40 ± 0.01, 10 mM Hepes, 50 mM NaCl, 1 mM CaCl_2_, 1 mM MnCl_2_) which was used as running buffer and two startup cycles were performed. The experiment was performed in multi cycle mode. For all experiments a 1 mg/mL stock solution of Con A was prepared in LBB and was shaken for 1 h. Then it was filtrated (Acrodisc^®^ 32 mm syringe filter with a 0.1 µm Supor^®^ membrane from Pall Corporation, Newquay, UK) and the concentration was determined spectrophotometrically. The stock solution was diluted to give a 200 nM solution of Con A. Glyco-AuNP solutions were concentrated by centrifugation (Allegra^TM^ 25R Centrifuge, Beckman Coulter, Brea, CA, USA, 12 h, 4500 rcf) and were resuspended in LBB to give a 20 nM stock solution. The concentration was verified by UV measurement. This glyco-AuNP stock solution was then used to prepare the dilution series in LBB. For glyco-AuNP **4** a dilution series with concentrations of 0, 0.02, 0.1, 0.2, 0.6, 0.8, 1, 1.5, 2 nM, for **5** 0, 0.02, 0.1, 0.2, 0.4, 0.6, 0.8, 1, 1.5, 2 nM and for **6** 0, 0.02, 0.1, 0.2, 0.4, 0.6, 0.8, 1, 1.5, 2 nM were prepared. For *N*-ethylmaleimide capped glycomacromolecule **1a** a dilution series with concentrations of 0, 1, 10, 20, 50, 100, 200, 500, 1000 µM and **3a** a dilution series with concentrations of 0, 2, 20, 100, 200, 500, 1000, 1500, 2000 nM was prepared. For αMeMan a dilution series with concentrations of 0, 10, 100, 500, 1000, 1500, 2000, 5000 µM was prepared. As negative control, four representative concentrations of 0, 10, 1500 and 5000 µM were prepared for d-(+)-galactose.

For the experiment Con A and sample solutions were mixed in a 1:1 ratio to give half of the prepared concentrations. Each mixture was injected over both flow cells whereas the binding signal on flow cell 1 was subtracted from the response on flow cell 2. As regeneration procedure three consecutive injections of a 0.8 M solution of αMeMan in LBB with a contact time of 30 s was used. The relative response value at the beginning of the dissociation phase (20 s after the end of sample injection) for the 100 nM Con A solution with zero sample concentration was set to 100% binding. The relative response values of the Con A solutions incubated with the respective sample in serial dilutions were referred to Con A and calculated for relative binding in %. IC_50_ values were calculated as concentration of the sample that result in 50% inhibition of Con A binding to the mannosylated sensor chip surface. RIP (relative inhibitory potency) values are based on the IC_50_ value for αMeMan (IP (inhibitory potency) ≡ 1). All glyco-AuNPs, glycomacromolecules and αMeMan have been tested in three independent experiments (triplicate determination per experiment for αMeMan and glyco-AuNPs, dublicate determination per experiment for glycomacromolecules **1a** and **3a**).

### 2.17. Syntheses

#### 2.17.1. Syntheses of Building Blocks:

The method employed for the syntheses of the TDS functional building block as well as the EDS spacer building block followed the procedures reported previously by Hartmann and coworkers [54,81].

##### 1-(Fluorenyl)-3,11-dioxo-7-(pent-4-ynoyl)-2-oxa-4,7,10-triazatetradecan-14-oic acid, TDS

The overall yield of the synthesis was 23%. ^1^H-NMR (600 MHz, DMSO-*d*_6_): δ(ppm) = 11.84 (br s, –OH), 8.03 (t, ^3^*J*_HH_ = 5.75 Hz, –NH), 7.87 (d, ^3^*J*_HH_ = 7.55 Hz, 2H, Ar–H), 7.67 (dd, ^3^*J*_HH_ = 7.36, ^4^*J*_HH_ = 2.47 Hz, 2H, Ar–H), 7.43 (t, ^3^*J*_HH_ = 7.44 Hz, 3H, Ar–H),7.39 (t, ^3^*J*_HH_ = 5.93 Hz, –N–H), 7.37–7.30 (m, 2H, Ar–H), 4.30 (d, ^3^*J*_HH_ = 6.82 Hz, 1H, –C(O)OC**H**_2_CH–), 4.27 (d, ^3^*J*_HH_ = 6.94 Hz, 1H, –C(O)OC**H**_2_CH–), 4.21 (t, ^3^*J*_HH_ = 6.79 Hz, 1H, –C(O)OCH_2_C**H**–), 3.34–3.22 (m, 4H, –NC**H**_2_CH_2_NH–), 3.21–3.06 (m, 4H, –NCH_2_C**H**_2_NH–), 2.78–2.70 (m, 1H, –C(O)CH_2_CH_2_CC**H**), 2.54–2.47 (m, 2H, –C(O)C**H**_2_CH_2_CCH, overlapped by DMSO-*d*_5_), 2.46–2.38 (m, 2H, –C(O)CH_2_C**H**_2_CCH), 2.37–2.25 (m, 4H, –C(O)C**H**_2_C**H**_2_COOH). ESI-MS: *m/z* calcd. for C_28_H_31_N_3_O_6_ [M + H]^+^ 506.2; found 506.2.

##### 1-(9H-Fluoren-9-yl)-3,14-dioxo-2,7,10-trioxa-4,13-diazaheptadecan-17-oic acid, EDS 

The overall yield of the synthesis was 59%. ^1^H-NMR (600 MHz, DMSO-*d*_6_): δ(ppm) = 11.86 (br s, –OH), 7.94–7.85 (m, 3H, –NH, Ar–H), 7.69 (d, ^3^*J*_HH_ = 7.46 Hz, 2H, Ar–H), 7.41 (t, ^3^*J*_HH_ = 7.46 Hz, 2H, Ar–H), 7.33 (t, ^3^*J*_HH_ = 7.50 Hz, 2H, Ar–H), 4.30 (d, ^3^*J*_HH_ = 6.97 Hz, 2H, –C(O)OC**H**_2_CH–), 4.21 (t, ^3^*J*_HH_ = 6.92 Hz, 1H, –C(O)OCH_2_C**H**–), 3.50 (s, 4H, –OC**H**_2_C**H**_2_O–), 3.44–3.35 (m, 4H, –NHCH_2_C**H**_2_O–), 3.19 (q, ^3^*J*_HH_ = 5.82 Hz, 2H, –NHC**H**_2_CH_2_O–), 3.14 (q, ^3^*J*_HH_ = 5.87 Hz, 2H, –NHC**H**_2_CH_2_O–), 2.42 (t, ^3^*J*_HH_ = 6.72 Hz, 2H, –C(O)CH_2_C**H**_2_COOH), 2.32 (t, ^3^*J*_HH_ = 7.22 Hz, 2H, –C(O)C**H**_2_CH_2_COOH). ESI-MS: *m/z* calcd. for C_25_H_30_N_2_O_7_ [M + H]^+^ 471.2; found 471.2, [M + Na]^+^ 493.2, found 493.2.

##### Synthesis of ODS

The synthesis of the ODS building block follows the synthesis of the previously introduced EDS building block [54,81].

##### *N*^1^-Trityloctan-1,8-diamine

25.25 g (175 mmol) 1,8-diaminooctane were dissolved in 430 mL DCM under inert atmosphere. The solution was cooled to 0 °C. A solution of 12.2 g (43.75 mmol) tritylchloride in 250 mL DCM was added dropwise over a period of 1.5 h. The slurry white mixture was stirred overnight at room temperature. The reaction mixture was concentrated under reduced pressure. The organic phase was washed with saturated aqueous NaHCO_3_ solution (4 × 400 mL). The collected organic phases were dried over Na_2_SO_4_, filtered and the solvent was removed under reduced pressure to give 16.9 g (43.75 mmol) of a slightly yellow oil. The product was used directly in the next step without further purification. ^1^H-NMR (300 MHz, CDCl_3_): δ(ppm) = 7.52–7.46 (m, 6H, Ar–H), 7.32–7.24 (m, 6H, Ar–H), 7.17 (tt, ^3^*J*_HH_ = 7.21, ^4^*J*_HH_ = 1.29 Hz, 3H, Ar–H), 2.70 (t, ^3^*J*_HH_ = 7.21 Hz, 2H, NH_2_C**H**_2_–), 2.10 (t, ^3^*J*_HH_ = 7.01 Hz, 2H, –NHC**H**_2_–), 1.55–1.38 (m, 4H, –NHCH_2_C**H**_2_(CH_2_)_4_CH_2_C**H**_2_NH_2_), 1.38–1.12 (br s, 8H, –NH(CH_2_)_2_(C**H**_2_)_4_(CH_2_)_2_NH_2_). ESI-MS: *m/z* calcd. for C_27_H_34_N_2_ [M + H]^+^ 387.3; found 387.1.

##### (9H-Fluoren-9-yl)methyl (8-(tritylamino)octyl)carbamate

To a solution of 16.9 g (43.7 mmol) of N1-trityloctan-1,8-diamine in 220 mL THF, 11.3 g (43.7 mmol) Fmoc chloride and 30.2 g (218.5 mmol) K_2_CO_3_ in 220 mL water were added and stirred vigorously overnight. THF was removed under reduced pressure. The remaining slightly yellow oil on the water phase was redissolved in 300 mL ethyl acetate. The organic phase was washed with water (3 × 300 mL), dried over Na_2_SO_4_, filtered and the solvent was removed under reduced pressure to give 26.6 g (43.7 mmol) of a yellow oil. The product was used directly in the next step without further purification. ^1^H-NMR (300 MHz, CDCl_3_): δ(ppm) = 7.80–7.74 (m, 2H, Ar–H), 7.05–7.55 (m, 2H, Ar–H), 7.54–7.42 (m, 6H, Trt–H), 7.42–7.35 (m, 2H, Ar–H), 7.34–7.23 (m, 8H, Trt–H, Ar–H), 7.22–7.14 (m, 3H, Trt–H), 4.40 (d, ^3^*J*_HH_ = 6.86 Hz, 2H, –C(O)OC**H**_2_CH–), 4.22 (t, ^3^*J*_HH_ = 6.79 Hz, 1H, –C(O)OCH_2_C**H**–), 3.17 (m, 2H, –NHC**H**_2_–), 2.11 (m, 2H, –NHC**H**_2_–), 1.54–1.38 (m, 4H, –NHCH_2_C**H**_2_–), 1.37–1.12 (m, 8H, –NH(CH_2_)_2_(C**H**_2_)_4_(CH_2_)_2_NH–). ESI-MS: *m/z* calcd. for C_42_H_44_N_2_O_2_ [M + H]^+^ 609.3; found 609.3.

##### 8-((((9H-Fluoren-9-yl)methoxy)carbonyl)amino)octan-1-aminium trifluoracetate 

To a solution of 26.6 g (43.7 mmol) of (9H-fluoren-9-yl)methyl (8-(tritylamino)octyl)carbamate in 300 mL DCM, 13.9 mL TES (87.4 mmol) and 30 mL TFA (10%) were added slowly. The reaction mixture was stirred at room temperature. After 2 h 300 mL toluene were added and TFA was coevaporated under reduced pressure to yield a light yellow oil. The oil was dissolved in 40 mL DCM and precipitated in Et_2_O in 8 small fractions. A white solid precipitated that was washed with Et_2_O twice. The Et_2_O was decanted and the colorless solid was dried under high vacuum to give 18.6 g (40 mmol, 91%) of the product. ^1^H-NMR (300 MHz, CDCl_3_): δ(ppm) = 7.9 (br s, NH_3_^+^), 7.75 (d, ^3^*J*_HH_ = 7.43 Hz, 2H, Ar–H), 7.57 (d, ^3^*J*_HH_ = 7.43 Hz, 2H, Ar–H), 7.38 (t, ^3^*J*_HH_ = 7.31 Hz, 2H, Ar–H), 7.29 (t, ^3^*J*_HH_ = 7.17 Hz, 2H, Ar–H), 4.37 (d, ^3^*J*_HH_ = 6.67 Hz, 2H, –C(O)OC**H**_2_CH–), 4.19 (t, ^3^*J*_HH_ = 6.49 Hz, 1H, -C(O)OCH_2_C**H**–), 3.13 (br s, 2H, NH_3_C**H**_2_–), 2.93 (br s, 2H, –NHC**H**_2_–), 1.75–1.16 (m, 12H, –NH(CH_2_)(C**H**_2_)_6_(CH_2_)NH_3_^+^). ESI-MS: *m/z* calcd. for C_23_H_31_N_2_O_2_ [M]^+^ 367.2; found 367.5; [M + H]^+^ 368.24638; found 368.5.

##### 4-((8-((((9H-Fluoren-9-yl)methoxy)carbonyl)amino)octyl)amino)-4-oxobutanoic acid, ODS 

12.5 g (27 mmol) of the TFA salt precursor were dissolved in 300 mL DCM after the addition of 11 mL (81 mmol) triethylamine. 2.7 g (27 mmol) succinic anhydride were added to the colorless solution. The reaction mixture was stirred at room temperature. Some of the colorless product precipitated after 1 h. After 2 h the mixture was poured into 1 L of 5% aqueous solution of citric acid. Vigorous stirring leads to precipitation of a colorless fluffy solid. The solid was filtered and washed with 5% aqueous solution of citric acid and water. After drying the precipitate under vacuum it was redissolved in a DCM/MeOH/AcOH (100/10/1, *v/v*) mixture and was washed with water. In the second washing step a fluffy colorless precipitate was formed that is filtered and dried under high vacuum to yield 11.5 g (25 mmol, 92%) of the product. The overall yield of the synthesis was 85%. ^1^H-NMR (600 MHz, DMSO-*d*_6_): δ(ppm) = 11.60 (br s, 1H, –OH), 7.88 (d, ^3^*J*_HH_ = 7.40 Hz, 2H, Ar–H), 7.79 (t, ^3^*J*_HH_ = 5.35 Hz, 1H, –NH), 7.68 (d, ^3^*J*_HH_ = 7.37 Hz, Ar–H), 7.41 (t, ^3^*J*_HH_ = 7.37 Hz, 2H, Ar–H), 7.32 (t, ^3^*J*_HH_ = 7.37 Hz, Ar–H), 7.25 (t, ^3^*J*_HH_ = 5.64 Hz, 1H, –NH), 4.29 (d, ^3^*J*_HH_ = 6.84 Hz, 2H, –C(O)OC**H**_2_CH–), 4.20 (t, ^3^*J*_HH_ = 6.65 Hz, 1H, –C(O)OCH_2_C**H**–), 3.00 (q, ^3^*J*_HH_ = 6.32 Hz, 2H, –NHC**H**_2_–), 2.96 (q, ^3^*J*_HH_ = 6.32 Hz, 2H, –NHC**H**_2_–), 2.41 (t, ^3^*J*_HH_ = 6.18 Hz, 2H, –C(O)C**H**_2_CH_2_COOH), 2.29 (t, ^3^*J*_HH_ = 6.46 Hz, 2H, –C(O)CH_2_C**H**_2_COOH), 1.45–1.30 (m, 4H, –NHCH_2_C**H**_2_–), 1.29–1.19 (br s, 8H, –NH(CH_2_)(C**H**_2_)_6_(CH_2_)NH–). ^13^C–NMR (600 MHz, DMSO–d_6_): δ(ppm) = 173.90 (s, –COOH), 170.73 (s, –C(O)NH–), 156.10 (s, –NHC(O)O–), 143.96 (s, Ar–C), 140.76 (s, Ar–C), 127.60 (s, Ar–CH_._), 127.04 (s, Ar–CH), 125.15 (s, Ar–CH), 120.12 (s, Ar-CH), 65.16 (s, Fmoc–CH_2_–), 46.82 (s, Fmoc–CH–), 40.23 (s, -CH_2_COOH), 38.55 (c, –CH_2_CONH–), 30.06 (s, –CH_2_NH–), 29.39 (s, –CH_2_NH–), 29.26 (–CH_2_–), 29.16 (–CH_2_–), 28.77 (–CH_2_–), 28.73 (–CH_2_–), 26.40 (–CH_2_–), 26.22 (–CH_2_–). HR-ESI-MS for C_27_H_34_N_2_O_5_ [M + H]^+^ calcd. 467.2540; found 467.2549, mass accuracy −1.9 ppm. RP-HPLC (linear gradient from 0 to 95% eluent B in 17 min at 25 °C): *t*_r_ = 12.3 min. Determined purity: 99%.

##### (2-Azidoethyl)-2,3,4,6-tetra-*O*-acetyl-α-d-mannopyranoside 

The synthesis proceeded according to literature [82]. The overall yield of the synthesis was 17%. ^1^H-NMR (300 MHz, CDCl_3_): δ(ppm) = 5.34 (dd, ^3^*J*_HH_ = 10.01, 3.44 Hz, 1H, H3), 5.29 (d, ^3^*J*_HH_ = 9.99 Hz, 1H, H4), 5.26 (dd, ^3^*J*_HH_ = 3.33, 1.72 Hz, 1H, H2), 4.86 (d, ^3^*J*_HH_ = 1.45 Hz, 1H, H1), 4.27 (dd, ^3^*J*_HH_ = 5.32 Hz, ^2^*J*_HH_ = 12.26 Hz, 1H, H6), 4.11 (dd, ^3^*J*_HH_ = 2.35 Hz, ^2^*J*_HH_ = 12.27 Hz, 1H, H6), 4.06–4.01 (m, 1H, H5), 3.89–3.83 (m, 1H, –OC**H**_2_CH_2_N_3_), 3.69–3.63 (m, 1H, –OC**H**_2_CH_2_N_3_), 3.52–3.40 (m, 2H, –OCH_2_C**H**_2_N_3_), 2.15 (s, 3H, –OCH_3_), 2.09 (s, 3H, –OCH_3_), 2.04 (s, 3H, –OCH_3_), 1.99 (s, 3H, –OCH_3_). ESI-MS: *m/z* calcd. for C_16_H_23_N_3_O_10_ [M + NH_4_]^+^ 435.2; found 435.3.

All solution-phase reactions were monitored by thin-layer chromatography (TLC) using MACHERY-NAGEL silica gel 60 F254 (0.20 mm thickness). The TLC plates were visualized with UV light and by ninhydrine or sugar staining followed by heating.

#### 2.17.2. Solid Phase Polymer Synthesis 

Precision glycomacromolecules were synthesized on solid support by stepwise assembly of different building blocks according to general coupling protocol and Fmoc cleavage protocol [52]. As building blocks, functional building blocks bearing an alkyne group (TDS) and spacer building blocks with either hydrophilic (EDS) or hydrophobic (ODS) properties were employed. After successful assembly of the desired number of building blocks, capping of the N-terminal primary amine group was performed [52]. The subsequent conjugation of the azide functionalized carbohydrates proceeded by general CuAAC protocol [52]. As last step, the product was cleaved from solid support using 30% TFA and 1% TIPS in DCM and was precipitated in diethyl ether. Subsequently, the product was redissolved in MeOH and carbohydrate deprotection was performed in solution using 0.1 M NaOMe in MeOH. After 2 h shaking the solution was neutralized with Amberlite IR-120 (hydrogen form) and the resin was filtered off. After solvent evaporation the product was isolated by lyophilization. All glycomacromolecules were purified by prep-RP-HPLC (see Section 2.4).

For the more hydrophilic structures **1** and **3** commercially available H-Gly-2-chlorotrityl PS resin was used for solid phase synthesis. For the more hydrophobic structure **2** commercially available Fmoc-Gly-Trt Tentagel resin was used. The batch sizes for synthesizing the glycomacromolecules were 0.1 mmol for structures **1** and **3** and 0.05 mmol for **2**.

All solid phase reactions were performed in 10 mL polypropylene reactors with a polyethylene frit (Multisyntech GmbH, Witten, Germany), closed at the bottom with a luer stopper from Multisyntech GmbH (Witten, Germany).

##### Man(2)-5-Gly(1)-EDS(3,4)-Cys(5), **1**

Compound **1** was obtained in a yield of 78% after purification by prep-RP-HPLC. The anomeric proton of mannose could not be examined in ^1^H-NMR due to the overlaying signal of the residual solvent. ^1^H-NMR (600 MHz, D_2_O): δ(ppm) = 8.27 (s, –NH), 7.88 (s, 1H, Ar–H), 4.66–4.62 (m, 2H, –OCH_2_C**H**_2_N–(side chain)), 4.46 (t, ^3^*J*_HH_ = 6.2 Hz, 1H, –C(O)C**H**(CH_2_SH)NH–(Cys)), 4.12–4.06 (m, 1H, –OC**H**_2_CH_2_N–(side chain)), 3.95–3.90 (m, 3H, –OC**H**_2_CH_2_N–(side chain), –C(O)C**H**_2_NH–(Gly)), 3.88–3.85 (m, 1H, Man–H), 3.77–3.72 (m, 1H, Man–H), 3.70–3.58 (m, 19H, –OC**H**_2_C**H**_2_O–(EDS), –NHCH_2_C**H**_2_O–(EDS), Man–H), 3.51–3.32 (m, 16H, –C(O)NHC**H**_2_C**H**_2_N–(TDS), –NHC**H**_2_CH_2_O–(EDS)), 3.06–2.98 (m, 3H, Man–H, –NC(O)CH_2_C**H**_2_–(side chain)), 2.91 (d, ^3^*J*_HH_ = 5.9 Hz, 2H, –C(O)CH(C**H**_2_SH)NH–(Cys)), 2.80 (t, ^3^*J*_HH_ = 7.3 Hz, 2H, –NC(O)C**H**_2_CH_2_–(side chain)), 2.60–2.46 (m, 12H, –C(O)C**H**_2_C**H**_2_C(O)–), 2.07 (s, 3H, –C(O)CH_3_). MALDI-TOF-MS for C_48_H_82_N_12_O_21_S: [M + Na]^+^ calcd. 1217.5, found 1217.6, [disulfide + Na]^+^ calcd. 2410.1, found 2410.2. HR-ESI-MS for C_48_H_82_N_12_O_21_S (exact monoisotopic mass 1194.5438): [M + 2H]^2+^ calcd. 598.2792, found 598.2792, mass accuracy ±0.0 ppm. RP-HPLC (linear gradient from 0 to 50% eluent B in 30 min at 25 °C): *t*_r_ = 10.6 min. Determined purity: 95%.

##### Man(2)-5-Gly(1)-ODS(3,4)-Cys(5), **2**

Compound **2** was obtained in a yield of 11% after purification by prep-RP-HPLC. The anomeric proton of mannose could not be examined in ^1^H-NMR due to the overlaying signal of the residual solvent. ^1^H-NMR (600 MHz, D_2_O): δ(ppm) = 8.40 (s, –NH), 7.88 (s, 1H, Ar–H), 4.66–4.62 (m, 2H, –OCH_2_C**H**_2_N–(side chain)), 4.41 (t, ^3^*J*_HH_ =6.3 Hz, 1H, –C(O)C**H**(CH_2_SH)NH–(Cys)), 4.12–4.06 (m, 1H, –OC**H**_2_CH_2_N–(side chain)), 3.95–3.90 (m, 1H, –OC**H**_2_CH_2_N–(side chain)), 3.88–3.85 (m, 1H, Man–H), 3.83–3.79 (m, 2H, –C(O)C**H**_2_NH–(Gly)), 3.77–3.71 (m, 1H, Man–H), 3.70– 3.58 (m, 3H Man–H), 3.51–3.31 (m, 8H, –C(O)NHC**H**_2_C**H**_2_N–(TDS)), 3.28–3.10 (m, 8H, –NHC**H**_2_(CH_2_)_6_C**H**_2_NH–(ODS)), 3.07–2.97 (m, 3H, Man–H, –NC(O)CH_2_C**H**_2_–(side chain)), 2.89 (dd, *J*_HH_ = 1.8, 6.3 Hz, 2H, –C(O)CH(C**H**_2_SH)NH–(Cys)), 2.80 (t, ^3^*J*_HH_ = 7.2 Hz, 2H, –NC(O)C**H**_2_CH_2_–(side chain)), 2.61–2.44 (m, 12H, –C(O)C**H**_2_C**H**_2_C(O)–), 2.07 (s, 3H, –C(O)CH_3_), 1.53–1.43 (m, 8H, –NHCH_2_C**H**_2_(CH_2_)_4_C**H**_2_CH_2_NH–(ODS)), 1.33–1.22 (m, 16H, –NH(CH_2_)_2_(C**H**_2_)_4_(CH_2_)_2_NH–(ODS)). MALDI-TOF-MS for C_52_H_90_N_12_O_17_S: [M + Na]^+^ calcd. 1209.6, found 1209.6, [disulfide + Na]^+^ calcd. 2394.2, found 2396.3. HR-ESI-MS for C_52_H_90_N_12_O_17_S (exact monoisotopic mass 1186.6268): [M + 2H]^2+^ calcd. 594.3207, found 594.3202, mass accuracy −0.8 ppm. RP-HPLC (linear gradient from 0 to 50% eluent B in 30 min at 25 °C): *t*_r_ = 17.6 min. Determined purity: 89% (8% thioether side product).

##### Man(2,4,6,8,10)-11-Gly(1)-EDS(3,5,7,9)-Cys(11), **3**

Compound **3** was obtained in a yield of 34% after purification by prep-RP-HPLC. The anomeric proton of mannose could not be examined in ^1^H-NMR due to the overlaying signal of the residual solvent. For the acetyl group on the N-terminal end two singlets with a 1:1 ratio were observed that we assign to two rotational isomers in equal amounts. ^1^H-NMR (600 MHz, D_2_O): δ(ppm) = 8.32 (s, –NH), 7.89–7.86 (m, 5H, Ar–H), 4.66–4.62 (m, 10H, –OCH_2_C**H**_2_N–(side chain)), 4.45–4.40 (m, 1H, –C(O)C**H**(CH_2_SH)NH– (Cys)), 4.12–4.06 (m, 5H, –OC**H**_2_CH_2_N–(side chain)), 3.94–3.84 (m, 12H, –OC**H**_2_CH_2_N–(side chain), –C(O)C**H**_2_NH–(Gly), Man–H), 3.76–3.72 (m, 5H, Man–H), 3.70–3.57 (m, 47H, –OC**H**_2_C**H**_2_O–(EDS), –NHCH_2_C**H**_2_O–(EDS), Man–H), 3.52–3.29 (m, 56H, –C(O)NHC**H**_2_C**H**_2_N–(TDS), –NHC**H**_2_CH_2_O–(EDS)), 3.06–2.96 (m, 15H, Man–H, –NC(O)CH_2_C**H**_2_–(side chain)), 2.90–2.83 (m, 2H, –C(O)CH(C**H**_2_SH)NH–(Cys)), 2.80 (t, ^3^*J*_HH_ = 7.1 Hz, 10H, –NC(O)C**H**_2_CH_2_–(side chain)), 2.60–2.44 (m, 36H, –C(O)C**H**_2_C**H**_2_C(O)–), 2.06 + 2.04 (2s, 3H, –C(O)CH_3_). MALDI–TOF–MS for C_152_H_254_N_40_O_65_S: [M + Na]^+^ calcd. 3734.7, found 3734.8. HR-ESI-MS for C_152_H_254_N_40_O_65_S (exact monoisotopic mass 3711.7520): [M + 4H]^4+^ calcd. 928.9453, found 928.9450, mass accuracy −0.3 ppm. RP-HPLC (linear gradient from 0 to 50% eluent B in 30 min at 25 °C): *t*_r_ = 11.9 min. Determined purity: 95%.

#### 2.17.3. Capping of Glycomacromolecule-Thiol Functionalities with *N*-ethylmaleimide

For capping of the thiol functionalities the respective glycomacromolecule was dissolved in degassed buffer (10 mM Hepes, pH 7) in a concentration of 5 mM. 10 eq. of Tris(2-carboxyethyl)phosphine hydrochloride (TCEP) were added under nitrogen stream. The reaction mixture was shaken for 48 h. Then 15 eq. of *N*-ethylmaleimide were added under nitrogen stream and the reaction was shaken for further 12 h at room temperature. Glycomacromolecules **1a** and **3a** were purified by preparative HPLC (H_2_O with 0.1% formic acid (A) and MeCN with 0.1% formic acid (B); 95% A to 5% A in 10 min).

##### Man(2)-5-Gly(1)-EDS(3,4)-Cys(5)-*N*-ethylmaleimide, **1a**

Compound **1a** was obtained in a yield of 73% after preparative HPLC. The anomeric proton of mannose could not be examined in ^1^H-NMR due to the overlaying signal of the residual solvent. For the acetyl group on the N-terminal end two singlets with a 1:1 ratio were observed in ^1^H-NMR that were assigned to two rotational isomers in equal amounts. ^1^H-NMR (300 MHz, D_2_O): δ(ppm) = 8.28 (s, –NH), 7.88 (s, 1H, Ar–H), 4.67–4.61 (m, 2H, –OCH_2_C**H**_2_N–(side chain)), 4.61–4.53 (m, 1H, –C(O)C**H**(CH_2_S)NH–(Cys)), 4.14–4.01 (m, 2H, –OC**H**_2_CH_2_N–(side chain), –C(O)CH(CH_2_SC**H**)NH–(*N*-ethylmaleimide)), 3.97–3.83 (m, 4H, –OC**H**_2_CH_2_N–(side chain), –C(O)C**H**_2_NH–(Gly), Man–H), 3.78–3.71 (m, 1H, Man–H), 3.71–3.24 (m, 37H, –OC**H**_2_C**H**_2_O–(EDS), –NHCH_2_C**H**_2_O–(EDS), Man–H, –C(O)NHC**H**_2_C**H**_2_N–(TDS), –NHC**H**_2_CH_2_O–(EDS), –NC**H**_2_CH_3_(*N*-ethylmaleimide)), 3.17 (d, ^3^*J*_HH_ =6.7 Hz, 1H, –C(O)CH(C**H**_2_S)NH–(Cys)), 3.08–2.96 (m, 3H, Man–H,–NC(O)CH_2_C**H**_2_–(side chain)), 2.80 (t, ^3^*J*_HH_ = 7.2 Hz, 2H, –NC(O)C**H**_2_CH_2_–(side chain)), 2.72 (t, ^3^*J*_HH_ = 4.1 Hz, 1H, –SCHCH_2_–(*N*-ethylmaleimide)), 2.65 (t, ^3^*J*_HH_ = 4.1 Hz, 1H, –SCHC**H**_2_–(*N*-ethylmaleimide)), 2.63–2.44 (m, 12H, –C(O)C**H**_2_C**H**_2_C(O)–), 2.07 + 2.06 (2s, 3H, –C(O)CH_3_), 1.13 (t, ^3^*J*_HH_ = 7.3 Hz, 3H, NCH_2_C**H**_3_ (*N*-ethylmaleimide)). MALDI-TOF-MS for C_54_H_89_N_13_O_23_S: [M + Na]^+^ calcd. 1342.6, found 1342.6. HR-ESI-MS for C_54_H_89_N_13_O_23_S (exact monoisotopic mass 1319.5915): [M + 2H]^2+^ calcd. 660.8030, found 660.8024, mass accuracy −0.9 ppm. RP-HPLC (linear gradient from 0 to 50% eluent B in 30 min at 25 °C): *t*_r_ = 12.0 min. Determined purity: 97%.

##### Man(2,4,6,8,10)-11-Gly(1)-EDS(3,5,7,9)-Cys(11)-*N*-ethylmaleimide, **3a**

Compound **3a** was obtained in a yield of 44% after preparative HPLC. The anomeric proton of mannose could not be examined in ^1^H-NMR due to the overlaying signal of the residual solvent. For the acetyl group on the N-terminal end two singlets with a 1:1 ratio were observed that were assigned to two rotational isomers in equal amounts. ^1^H-NMR (300 MHz, D_2_O): δ(ppm) = 7.88 (s, 5H, Ar–H), 4.67–4.60 (m, 10H, -OCH_2_C**H**_2_N–(side chain)), 4.58–4.49 (m, 1H, –C(O)C**H**(CH_2_SCH)NH–(Cys)), 4.14–4.04 (m, 5H, -OC**H**_2_CH_2_N–(side chain)), 4.04–3.98 (m, 1H, –C(O)CH(CH_2_SC**H**)NH–(*N*-ethylmaleimide)), 3.97–3.84 (m, 13H, –OC**H**_2_CH_2_N–(side chain), –C(O)C**H**_2_NH–(Gly), Man–H), 3.78–3.71 (m, 5H, Man–H), 3.70–3.55 (m, 47H, –OC**H**_2_C**H**_2_O–(EDS), –NHCH_2_C**H**_2_O–(EDS), Man–H), 3.52–3.30 (m, 58H, –C(O)NHC**H**_2_C**H**_2_N–(TDS), –NHC**H**_2_CH_2_O–(EDS), –NC**H**_2_CH_3_ (*N*-ethylmaleimide)), 3.17–3.09 (m, 2H, –C(O)CH(C**H**_2_S)NH–(Cys)), 3.07–2.95 (m, 15H, Man–H, –NC(O)CH_2_C**H**_2_–(side chain)), 2.80 (t, ^3^*J*_HH_ = 7.2 Hz, 10H, -NC(O)C**H**_2_CH_2_–(side chain)), 2.72–2.60 (m, 2H, –SCHC**H**_2_–(*N*-ethylmaleimide)) 2.55–2.42 (m, 36H, –C(O)C**H**_2_C**H**_2_C(O)–), 2.06 + 2.04 (2s, 3H, –C(O)CH_3_), 1.11 (td, ^3^*J*_HH_ = 1.6, 7.3 Hz, 3H, NCH_2_C**H**_3_ (*N*-ethylmaleimide)). MALDI-TOF-MS for C_158_H_261_N_41_O_67_S: [M + Na]^+^ calcd. 3859.8, found 3859.7. HR-ESI-MS for C_158_H_261_N_41_O_67_S (exact monoisotopic mass 3836.7997): [M + 4H]^4+^ calcd. 960.2072, found 960.2065, mass accuracy −0.7 ppm. RP-HPLC (linear gradient from 0–50% eluent B in 30 min at 25 °C): *t*_r_ = 12.5 min. Determined purity: 98%.

#### 2.17.4. Synthesis of Citrate Stabilized AuNPs

Spherical citrate stabilized AuNPs were synthesized according to the well-known Turkevich-method [83]. Briefly, 545 mL of water and 5.46 mL of HAuCl_4_ (0.05 M in water) were brought to the boil. 27.5 mL of a hot aqueous solution of sodium citrate dihydrate (1 wt %) were added rapidly under vigorous stirring. After the reduction was completed the dark red nanoparticle dispersion was allowed to cool to room temperature. According to TEM-analysis the AuNPs are spherical and monodisperse with a diameter of 14.1 ± 1.2 nm (see Figures S17 and S18).

#### 2.17.5. Functionalization of AuNPs by Ligand Exchange

All glassware that was used for the ligand exchange experiments was washed with aqua regia and MilliQ water. 100 mL of citrate stabilized AuNPs (5.5 × 10^−10^ mol) were transferred into a 250 mL round bottom flask. With the assumption that one glycomacromolecule occupies 1 nm² of the AuNP surface, 200% excess of glycomacromolecule (6.85 × 10^−7^ mol) was used. The glycomacromolecule was dissolved in MilliQ water (1 mg/mL) and preactivated with 2 eq. of TCEP. The AuNP solution was stirred vigorously while preactivated glycomacromolecule solution was added dropwise. The stirring speed was reduced, and the reaction mixture was stirred at room temperature in the dark for 48 h. Then, the reaction mixture was transferred into 50 mL falcon tubes and purified by three centrifugation steps (Allegra^TM^ 25R Centrifuge, Beckman Coulter, Brea, CA, USA, 2000× *g*, 20 °C, overnight) and each time redispersed in water. After centrifugation, glyco-AuNP concentration was determined spectrophotometrically (**4**: 42.6 nM, **5**: 26.0 nM, **6**: 37.7 nM).

## 3. Results and Discussion

### 3.1. Synthesis of Thiol Functionalized Precision Glycomacromolecules

**Figure 1:**
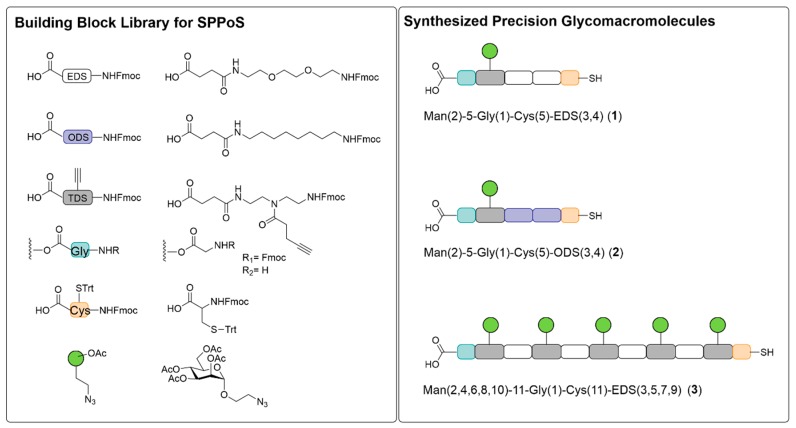
Schematic presentation of the building block library used for SPPoS and of the three synthesized glycomacromolecular structures **1**–**3**.

Thiol functionalized precision glycomacromolecules were synthesized employing previously introduced solid phase polymer synthesis and tailor-made building blocks [52,53,54,60,62]. In short, all building blocks are equipped with a terminal carboxy and a terminal Fmoc-protected amine group allowing for solid phase assembly via well-established Fmoc-peptide coupling protocols. Here we employ previously developed EDS and TDS building blocks, introducing ethylene glycol spacer units within the backbone and alkyne side chains for later sugar conjugation in the side chains (Figure 1). Furthermore, direct combination with Fmoc amino acid derivatives is possible and allows for the straightforward introduction of additional functional groups. For attachment of the glycomacromolecules to AuNPs, here we introduce a l-cysteine as final building block to give a terminal thiol group for later ligand exchange functionalization. As first building block, a glycine was introduced by employing a pre-loaded resin giving a free carboxy group at the other chain end that should support colloidal stability of the final glyco-AuNPs by electrostatic stabilization. In addition, a novel spacer building block is used for the first time, the so-called ODS building block. As starting material for the synthesis of this more hydrophobic aliphatic spacer building block, 1,8-diaminooctane was applied. In the following steps, the Fmoc group and the carboxyl functionality required for application in solid phase synthesis were introduced according to the established EDS synthesis [54,81]. Successful ODS synthesis was confirmed by HPLC, MS and ^1^H- and ^13^C-NMR analysis. A first set of macromolecular scaffolds was assembled varying the backbone composition by using either ODS or EDS spacer building blocks and the number of TDS building blocks from one to five. Copper catalyzed azide alkyne cycloaddition (CuAAC) of α-mannopyranoside-azide derivative following previously established conjugation protocols [54] gave the final set of thiol functionalized glycomacromolecules (**1**–**3**) (Figure 1).

All three glycomacromolecules exhibit a thiol functionality on the one end and a carboxyl functionality on the other end of the glycomacromolecule. Both, compounds **1** and **2** are monovalent in regard to their carbohydrate valency but exhibit different backbone properties with an ethylene glycol spacer for **1** and an aliphatic spacer for **2**. Compound **3** exhibits a higher valency with five carbohydrate ligands that are spaced by the hydrophilic EDS building block. For both monovalent structures **1** and **2** two spacer building blocks are installed in between the carbohydrate carrying building block TDS and the terminal l-cysteine to increase the overall chain length of the monovalent macromolecules and thereby potentially enhance sterical stabilization of glyco-AuNP after functionalization.

For glycomacromolecule **2**, the sugar deprotection step in solution was critical as a thioether side product was formed as result of basic abstraction of the Cα proton of l-cysteine followed by sulfur extrusion. The obtained thiolate anion then can react with the alkene functionality generating a thioether [84]. Interestingly, this side product formation was only observed for glycomacromolecules when employing the aliphatic spacer building block ODS. The thioether formation was observed even after purification by preparative HPLC (see Figures S1 and S2). Due to the fact that the thioether should not interfere with the ligand exchange reaction on the AuNPs, it was decided to use compound **2** without further purification. All glycomacromolecular structures were confirmed by HRMS, HPLC and ^1^H-NMR (see Figures S1–S10).

### 3.2. Preparation and Characterization of Glyco-AuNPs

**Figure 2:**
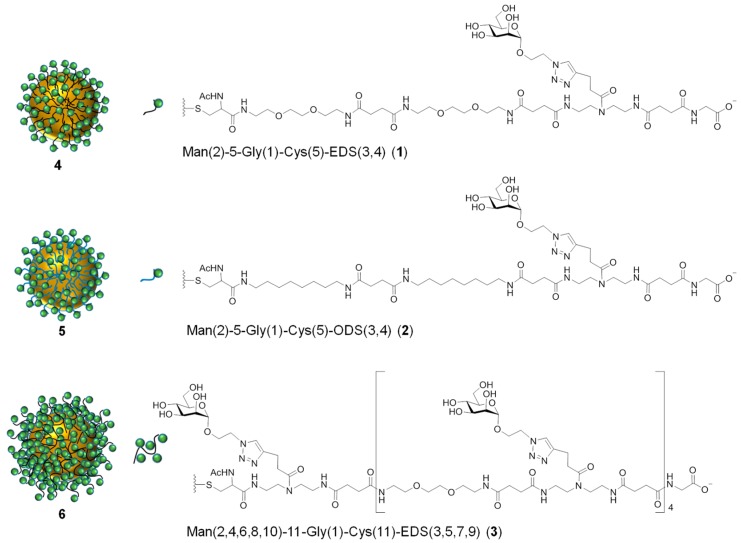
Chemical structure of precision glycomacromolecules **1**–**3** and schematic presentation of the associated glyco-AuNPs **4**–**6**.

Accordingly, three glyco-AuNPs **4**–**6** (Figure 2) were prepared following established protocols for ligand exchange reactions [85] employing the glycomacromolecules **1**–**3**. In short, the initial synthesis of citrate stabilized AuNPs was followed by the exchange of the temporary ligands by thiol functionalized precision glycomacromolecules. Subsequently, the obtained glyco-AuNPs were purified by several consecutive centrifugation steps and resuspension in MilliQ water.

The final compounds were characterized by UV-Vis absorbance spectroscopy and dynamic light scattering (DLS) (see Figures S19 and S20). The absorbance spectra show single peaks with small width, characteristic for the dipolar LSPR of small spherical AuNPs with low polydispersity (see Figure S19). A closer look reveals a maximum of the LSPR peak at approximately 517 nm for the citrate stabilized AuNPs and significantly redshifted LSPR peaks at approximately 521 nm for the glyco-AuNP **4** and 522 nm for the glyco-AuNPs **5** and **6**. The redshift of the LSPR upon ligand exchange by the glycomacromolecules is related to the increase of the local refractive index in the close vicinity of the AuNPs. Importantly the LSPR width did not change significantly after the ligand exchange and the absorbance quickly drops to zero at wavelengths higher than the LSPR maximum approaching the IR range. This indicates that the glyco-AuNPs are well stabilized and completely dispersed. In contrast to the observed behavior, the presence of aggregates would cause a significant broadening, shift and decrease in LSPR intensity due to near-field resonance coupling between AuNPs in close proximity. The intensity-time autocorrelation functions measured by DLS (see Figure S20) shows monomodal exponential decays as expected for the Brownian motion of colloidally stable, spherical colloids. The hydrodynamic radii of glyco-AuNPs **4**–**6** and citrate stabilized NPs in MilliQ water as obtained from second-order Cumulant analysis of all correlation functions are listed in Table 1. The larger hydrodynamic radii for **4**–**6** as compared to the citrate stabilized AuNPs are consistent with the functionalization of the AuNPs with glycomacromolecular structures **1**–**3**. Glyco-AuNPs **5** introducing the more hydrophobic spacer gives a larger hydrodynamic radius in comparison to the more hydrophilic system **4**, although both glycomacromolecules comprise of the same number of building blocks. This might be a first effect of the differences in macromolecular scaffold composition. Glyco-AuNPs **6** exhibit a larger hydrodynamic radius in comparison to glyco-AuNPs **4** as can be expected due to the longer chain length of the macromolecular scaffold.

The degree of functionalization for glyco-AuNPs was first determined based on the amount of carbohydrates per nanoparticle employing the phenol sulphuric acid method (PSA). Unfortunately, the absorbance at 490 nm for the carbohydrate-phenol-based dye formed during the assay overlaps with the absorbance of the AuNPs leading to large errors for this method (Table 1). Therefore, an additional method was employed. This method is based on the ionic interaction of the Toluidine Blue O (TBO) dye with the carboxylic end groups of the glycomacromolecules. Here, the absorbance at 633 nm is not impaired by the LSPR absorption of the AuNPs. The determined degree of glycomacromolecule functionalization for both methods is in good agreement (Table 1). From the determined degree of functionalization, an average degree of carbohydrate functionalization and occupied surface area can be calculated as given in Table 1. An occupied surface area of approximately 2 nm^2^ for all glycomacromolecular structures indicates good exchange of the initially stabilizing citrate molecules. Such dense surface coating is desirable for following binding studies with the model lectin Con A, as suggested by findings from Russell and coworkers showing that maximal aggregation with Con A is achieved with a monolayer of 100% mannose [38]. Glyco-AuNPs samples **5** and **6** show similar degree of functionalization of about 230 glycomacromolecules per nanoparticle, whereas sample **4** shows a slightly higher degree of functionalization with about 322 glycomacromolecules per nanoparticle (Table 1). As a result, the direct linkage of glycomacromolecule **3** to the gold surface where no additional spacer was installed between the first carbohydrate moiety and the terminal l-cysteine building block seems to have no effect on the degree of functionalization of the glyco-AuNP.

For the following binding studies with the model lectin Con A, sufficient stability of the synthesized glyco-AuNPs in lectin binding buffer (LBB) is critical. Therefore, glyco-AuNPs were first tested for their long-term stability in the presence of increasing sodium chloride concentrations attempting to exclude unsuitable glycomacromolecule candidates at an early stage of the experiments (see Figure S21). However, all glyco-AuNPs (**4**–**6**) exhibited high stability up to sodium chloride concentrations of 125 mM for 24 h, whereas the citrate stabilized AuNPs aggregated within seconds when exposed to sodium chloride concentrations higher than 25 mM. Thus, the employed glycomacromolecular scaffolds clearly increased AuNP stability under high salt concentrations. In the next step LBB composition (pH 7.40 ± 0.01, 10 mM Hepes, 50 mM NaCl, 1 mM CaCl_2_, 1 mM MnCl_2_) was tested for glyco-AuNP stability. The sodium chloride concentration lies within the permitted concentration limit but it was unknown how far additional salts could provoke unwanted aggregation of the glyco-AuNPs. Upon exposure of glyco-AuNPs to LBB no change in the SPR absorbance and the UV-Vis spectra were observed over a time range of 24 h (see Figure S21). Thus, our glyco-AuNPs meet the requirements for studying lectin binding. Furthermore, functionalization of AuNPs with precision glycomacromolecules seems to give highly stable glyco-AuNPs with high degree of functionalization and thus makes these a valuable platform for further investigations and potential applications.

### 3.3. Lectin Binding Studies with Model Lectin Con A

In the next step, we investigated the aggregation and binding behavior of the glyco-AuNPs upon exposure to Con A. Con A is used as model system since it is very well characterized and commercially available. It is known to specifically bind α-d-mannopyranosides [86,87,88,89,90] and in dependence on the pH, Con A has been shown to exist in different conformations. Under acidic pH it is present in a predominantly divalent conformation, while it forms tetramers in neutral buffer medium [89,90,91] as it is the case for the here applied LBB conditions.

In terms of the glyco-AuNP, we take into consideration two structural features: As we changed the glycomacromolecular backbone from an ethylene glycol spacer to an aliphatic spacer for glyco-AuNPs **4** and **5**, we compare these two compounds for a potential effect of the backbone composition on glyco-AuNP lectin interaction. Moreover, comparing results for glyco-AuNPs **4** and **6** can give first insights on how the valency and size of the glycomacromolecular structure affects lectin binding when presented on AuNPs.

**Figure 3:**
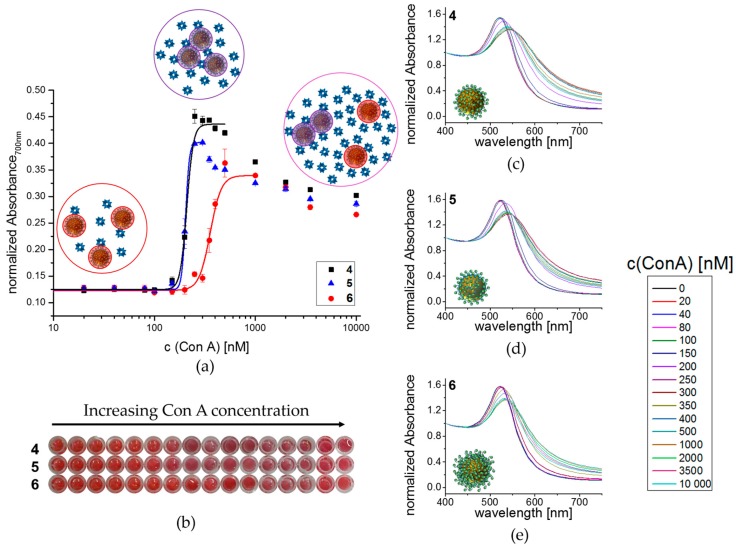
Aggregation behavior of glyco-AuNPs when exposed to different concentrations of Con A: (**a**) The normalized absorbance (normalized to absorbance at 400 nm) at 700 nm is plotted in dependence of Con A concentration, the different situations in solution are presented schematically; (**b**) Photograph of 96-well plate (background removed); (**c**) UV-Vis absorbance spectra for **4**; (d) **5**; (**e**) **6** in dependence of Con A concentration.

In the first employed assay, we looked at the aggregation behavior of glyco-AuNPs **4**–**6** in presence of increasing Con A concentration. Therefore, the glyco-AuNPs were added to a 96-well plate at final concentrations of 5 nM. Then Con A was added to the wells as a dilution series at final concentrations ranging from 20 nM to 10,000 nM and was incubated for 30 min before absorbance spectra were recorded. Similar assays have been presented before and were used in biosensor applications e.g., to detect different lectins by naked eye as a color change from red to purple/blue occurs upon aggregation (crosslinking) of glyco-AuNPs with lectins (Figure 3b) [35,38,39,40,41,42,43,44,45]. Spectrophotometrically, this color change is noted by a shift of the LSPR peak to longer wavelength and an increase in absorbance at 700 nm (Figure 3c–e) in the normalized (normalized to absorbance at 400 nm) UV-Vis spectra. By plotting the normalized absorbance (normalized to absorbance at 400 nm) at 700 nm against the Con A concentration, it was possible to obtain dose-dependent binding isotherms (Figure 3a). These binding isotherms show a constant, low absorbance at low Con A concentrations up to approximately 100 nM. Further increase in the Con A concentration leads to a steep increase in absorbance to approximately three times the absorbance in a concentration range of approximately 150 to 400 nM. This strong increase is related to the formation of aggregates, where the Con A bridges individual glyco-AuNPs. Within the aggregates AuNPs are distributed in close proximity and thus near-field plasmon resonance coupling occurs increasing the absorbance at 700 nm. After reaching a maximum value, the absorbances decrease continuously with a further increase of the Con A concentration. This decrease is related to a decreasing fraction of aggregated glyco-AuNPs and an increasing fraction of fully Con A saturated, individually dispersed glyco-AuNPs. Hence, the fraction number of aggregates decreases for increasing Con A concentrations causing a decrease of the absorbance at 700 nm. However, by fitting the Hill functions of the glyco-AuNPs up to plateau concentrations of Con A, the apparent dissociation constant *K*_D,app_ as a measure of the affinity of the different glyco-AuNPs towards Con A could be obtained (Table 2). For better comparability, *K*_D,app_ values that are corrected for the number of glycomacromolecules as well as the number of carbohydrate ligands per glyco-AuNP are also shown in Table 2.

The determined *K*_D,app_ values (Table 2) display that both monovalently decorated glyco-AuNPs **4** and **5** show similar affinities to Con A irrespective of their backbone composition. Since the degree of functionalization was slightly different for these two glyco-AuNPs (see Table 1), the normalized values differ with the more hydrophobic glycomacromolecule showing a higher normalized affinity. Since both parameters, hydrophilicity of the spacer as well as loading and thereby density of the carbohydrate ligands has been shown to influence lectin binding [45,71,73,74], potentially here both effects overlap. For example, Tsutsumi and coworkers found that gold nanoparticles conjugated with monosaccharide-modified peptides showed more than 10-fold higher aggregation activity when the hydrophobic amino acids tryptophan and phenylalanine compared to alanine surrounded the carbohydrate unit. They propose that the strong aggregation activity is caused by the interaction between hydrophobic amino acids near the carbohydrate binding site of Con A and the hydrophobic amino acid side chains in the synthesized peptide functionalized gold nanoparticles [45]. A similar behavior of the more hydrophobic spacer building block ODS compared to the hydrophilic spacer EDS could also explain the higher binding affinity of glyco-AuNPs **5** in the present study when normalized on the number of glycomacromolecules. However, further studies are required to investigate both parameters, hydrophobicity and degree of functionalization, separately, e.g., by a series of glyco-AuNPs with different degrees of functionalization. The glyco-AuNP **6** that is decorated with the longer and pentavalent structure **3** shows lower binding affinity (highest *K*_D,app_), especially when normalized on the number of carbohydrate ligands. Previously, we have observed that binding affinity of glycomacromolecules increases with the number of sugar ligands attached to the macromolecular scaffold [52,54]. However, we and others have also seen that when going to higher molecular weight compounds, most likely accessibility of the carbohydrate ligands is reduced resulting in an apparent decrease in lectin binding [52,71]. It should be noted again that the pentavalent structure **3** does not contain an additional EDS spacing between the anchoring l-cysteine moiety and the first sugar ligand. A decrease in accessibility of the sugar ligands might therefore be further promoted by the shorter linker between nanoparticle and first ligand moiety. Moreover, it has been shown in literature that shorter structures result in faster aggregation than longer structures and that the chain length has a profound effect on the sensitivity of the assay [38,42]. Due to the fact that shorter chain length allow for the formation of denser aggregates, the particles are in closer distance to each other resulting in a more intense purple color due to a stronger near-field resonance coupling [38]. Thus, the observed *K*_D,app_ values might rather be a measure for the kinetic behavior and a reflection of minimal glyco-AuNP distances than a measure for real lectin affinity so that the best biosensor might not necessarily be the best binder or inhibitor of lectin interactions. 

**Figure 4:**
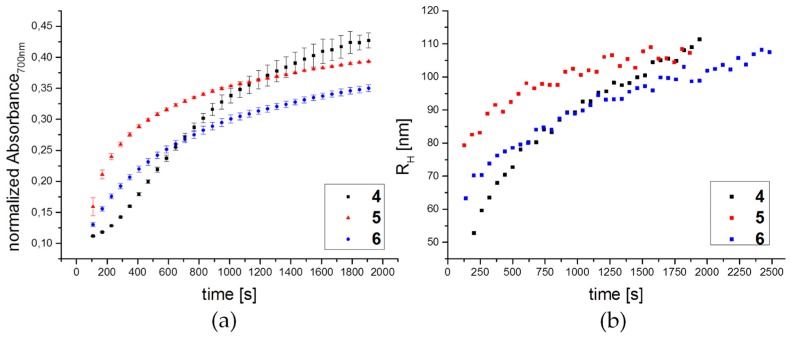
Kinetic analysis of glyco-AuNP interaction with ConA by (**a**) UV-Vis absorbance at 700 nm and (**b**) Representative evolutions of the hydrodynamic radii obtained from time-dependent DLS measurements at *K*_D,app_ concentrations of Con A.

To get insights into the aggregation kinetics as well as the origin of the pronounced color change from deep red to purple upon addition of Con A to the glyco-AuNPs, we performed time-dependent absorbance and DLS measurements. Figure 4a shows a continuous increase in absorbance at 700 nm for all three glyco-AuNPs **4**–**6** after addition of Con A at *K*_D,app_ concentration, added at time *t* = 0 s. In direct comparison, Figure 4b shows the evolution of the hydrodynamic radii R_H_ as a function of time. Apart from these representative curves obtained from single kinetic experiments, figure S21 shows results from three repetitive experiments for each system providing average values of *R*_H_ and the respective standard deviations. Relatively large error bars result from slight deviations in particle concentration and deviations in the mixing conditions. However, the general trend is clearly observable. All three samples show a continuous increase in *R*_H_. This increase indicates that aggregates are forming and grow with time. Consequently, the increase in absorbance at 700 nm in Figure 4a is related to the formation of aggregates with relatively small inter-particle distances allowing for near-field plasmon resonance coupling. Although pronounced differences in the evolution of *R*_H_ at short times up to about 500 s are observed, all three samples show a nearly linear increase in *R*_H_ with time at times larger than 500 s. In particular, the growth rate of the three samples seems to be very similar in this time regime. This could indicate that the cluster growth at larger times occurs independent of the type of glyco ligand and is most likely diffusion limited.

Unfortunately, a direct correlation between plasmon resonance coupling (color change) and aggregate size and in particular the aggregation number is not accessible from the present data. In particular, the change in absorbance at 700 nm is influenced by several parameters: (1) the average inter-particle distance within aggregated glyco-AuNPs depending on the ligand length that dictates the gap size between neighboring particles; (2) the fraction of aggregates with respect to dispersed glyco-AuNPs; (3) the average aggregation number. These quantities can only be determined quantitatively by small angle scattering techniques such as small angle X-ray scattering (SAXS). Such a study is outside the scope of the current manuscript and will be addressed in future work.

By addition of the free monosaccharide α-d-methyl-mannopyranoside (αMeMan) at an inhibitory concentration of 60 mM to the wells or cuvettes used for DLS, redispersion of the aggregates was observed (see Figure S22, Table 3). Moreover, control experiments with dilution series of BSA as non-carbohydrate binding protein in the 96-well format showed no color or spectral changes (see Figure S23). Both findings confirm that the observed aggregation was caused by specific carbohydrate-lectin interactions of the glyco-AuNP with Con A.

**Figure 5:**
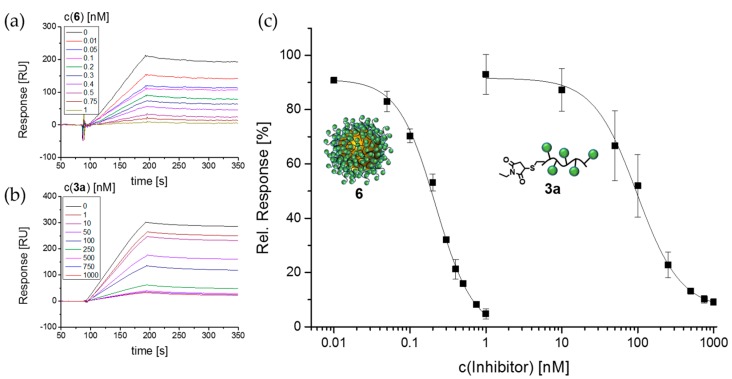
Exemplary SPR sensorgram for solutions of 100 nM Con A incubated with serial dilutions of (**a**) glyco-AuNP **6** and (**b**) *N*-methylmaleimide capped glycomacromolecule **3a**; (**c**) resulting IC_50_ curve fitted to Hill1 function for glyco-AuNP **6** and *N*-methylmaleimide capped glycomacromolecule **3a**.

Besides their use in biosensor applications [35,38,39,40,41,42,43,44,45], glyco-AuNPs have also been tested as inhibitors of lectin binding e.g., in the adhesion of pathogens [41,57,92]. In order to assess the potential of our glyco-AuNPs as inhibitors and whether the observed aggregation behavior also correlates with the inhibitory potency of glyco-AuNPs, an SPR inhibition competition assay [54,62] was employed. In the SPR experiment, a α-d-mannopyranoside functionalized polymer is immobilized on the sensor chip surface and inhibition curves are generated by measuring the response for 100 nM Con A in LBB in the presence of increasing concentrations of glyco-AuNPs **4**–**6** as competitive inhibitor (Figure 5). Due to the fact that the observed IC_50_ values may vary in dependence of the sensor chip surface, αMeMan was tested as inhibitor and well known reference on the same sensor chip to give the relative inhibitory potency (RIP) for comparison (see Figure S25). As a negative control for the assay setup d-(+)-galactose was measured in four representative concentrations which showed no inhibition (see Figure S26).

Furthermore, we directly compare the inhibitory potential of the glycomacromolecules and the respective glyco-AuNPs (Figure 5, Table 4, Figures S27–S30). Since the free thiol groups of the l-cysteine endgroups would lead to dimerization via disulfide formation, glycomacromolecules **1** and **3** were reacted with *N*-methylmaleimide to cap the free thiols (see Figures S11–S16). For glycomacromolecule **2** containing the ODS building block we observed dimerization via thioether formation as discussed above and therefore this structure was not used in the SPR study. Table 4 shows the determined IC_50_ and RIP values for capped glycomacromolecules **1a** and **3a** as well as for the glyco-AuNPs **4**–**6** in nM in comparison to the calculated RIP values corrected by the number of glycomacromolecules as well as carbohydrate ligands per nanoparticle. In agreement with previous findings [54], monovalent glycomacromolecule **1a** already shows an increase in the inhibitory potential in comparison to αMeMan. We attribute this finding to a sterical shielding effect where the non-binding parts of the glycomacromolecule block binding of competing ligands to the receptor. When increasing the valency as well as the overall size of the ligand, we see a further increase in the inhibitory potential. Interestingly, when comparing the single glycomacromolecules to the corresponding glyco-AuNP, we see a strong increase of about 400 times in the inhibitory potential per mannopyranoside for the monovalent system **1a** in comparison to glyco-AuNPs **4**. When going from the pentavalent ligand **3a** to the corresponding glyco-AuNPs **6**, we only see an increase in RIP per mannopyranoside of a factor of 3. Again this is in agreement with previous results, where we found that for glycomacromolecules with higher valency as well as higher molecular weights relative binding capacity per mannopyranoside stalls or even decreases [52,93]. So far we attribute this to a reduced accessibility of the ligands where not all mannopyranoside residues can take part in the protein binding. Nevertheless, glyco-AuNPs **6** show the lowest IC_50_ values. Interestingly, the inhibitory potential of the glyco-AuNPs **5** containing the more hydrophobic ODS building block is rather similar although the number of mannopyranoside ligands per AuNP is much lower (see Table 1). This finding correlates with our observation of the aggregation behavior of glyco-AuNPs as well as the *K*_D,app_ values and indicates an enhancement in protein binding through the more hydrophobic structure. However, further investigations are required to learn more about such potential hydrophobic interactions. 

Overall, the results from the inhibition competition study show the potential of increasing lectin binding when presenting glycomacromolecules on gold nanoparticles. Furthermore, we see that the structure of the glycomacromolecule strongly affects the resulting binding properties of the glyco-AuNPs. Thus, we believe that the combination of precision glycomacromolecules and AuNPs is an important tool to further study multivalent interactions.

## 4. Conclusions

We have shown the successful synthesis of a set of thiol-functionalized precision glycomacromolecules suitable for functionalization of AuNPs. Solid phase assembly of the glycomacromolecules allows for controlling the chemical structure and straightforward variation of different parameters such as the chemical composition of the scaffold and the number of presented carbohydrate ligands. The resulting glyco-AuNPs are highly functionalized and extremely stable under various salt and buffer conditions. Thus, they could be successfully implemented in a series of lectin binding studies using UV-Vis absorbance, DLS and SPR giving insights into the multivalent binding mechanisms of glyco-AuNPs. Future studies will extend on this concept by applying additional binding assays e.g., by performing hemagglutination or bacterial inhibition competition assays. Overall, we believe that the combination of precision glycomacromolecules and AuNPs is highly suitable for further investigations of structure-activity correlations in lectin binding but also for different applications e.g., as biosensors or inhibitors. 

## Figures and Tables

**Table 1:** The DLS derived hydrodynamic radii (*R*_H_) and degree of glycomacromolecule functionalization determined by phenol sulphuric acid method (PSA) and Toluidine Blue O-Titration (TBO) and derived average degree of carbohydrate functionalization and occupied surface area.

AuNP	*R*_H_ in Water [nm]	Degree of Glycomacromolecule Functionalization per NP (PSA)	Degree of Glycomacromolecule Functionalization per NP (TBO)	Average Degree of Carbohydrate Functionalization per NP	Average Occupied Surface Area per Glycomacro-Molecule [nm^2^]
Citrate stabilized	8.5 ± 0.1	-	-	-	-
4	11.7 ± 0.1	312 ± 218	332 ± 83	~322	~1.9
5	13.3 ± 0.3	231 ± 98	214 ± 57	~223	~2.8
6	13.5 ± 0.1	242 ± 2	243 ± 31	~1210	~2.5

**Table 2:** Apparent dissociation constant *K*_D,app_ in nM for glyco-AuNPs **4**–**6** and derived *K*_D,app_ values corrected for glycomacromolecule and carbohydrate number per nanoparticle in µM.

Glyco-Au-NP	*K*_D,app_ [nM]	*K*_D,app_ per Glycomacromolecular Ligand [µM]	*K*_D,app_ per Carbohydrate Ligand [µM]
4	209 ± 12	67	67
5	203 ± 4	45	45
6	357 ± 12	86	432

**Table 3:** DLS derived hydrodynamic radii (*R*_H_) for glyco-AuNPs in buffer medium as well as the determined R_H_ upon exposure to *K*_D,app_ concentration of Con A after 30 min and after subsequent addition of αMeMan. The values for the absorbance at wavelengths of 700 nm (normalized to absorbance at 400 nm) are an indirect measure for the particle aggregation. Error values for kinetic measurements refer to the root mean square deviation from at least three repetitive experiments.

Glyco-Au-NP	*R*_H_ in LBB [nm]	*R*_H_ at *K*_D,app_ conc. of Con A [nm]	*R*_H_ after αMeMan Addition [nm]	Normalized Abs_700 nm_ at *K*_D,app_ conc. of Con A
4	10.4 ± 0.2	106 ± 1	14.7 ± 0.7	0.43 ± 0.01
5	10.6 ± 0.1	112 ± 11	11.2 ± 0.2	0.39 ± 0.001
6	12.6 ± 0.1	101 ± 1	13.3 ± 0.2	0.35 ± 0.005

**Table 4:** IC_50_ and RIP values as measured for glyco-AuNPs **4**–**6** and *N*-ethylmaleimide capped glycomacromolecules **1a** and **3a** by SPR and derived RIP values corrected for glycomacromolecule and carbohydrate number per nanoparticle.

Glyco-Au-NP	IC_50_ [nM]	RIP	RIP per Glycomacromolecular Ligand	RIP per Carbohydrate Ligand
αMeMan	(5.17 ± 0.84) × 10^5^	1	1	1
1a ^1^	(0.7 ± 0.5) × 10^5^	5	5	5
3a ^1^	155 ± 78	2096	2096	419
4	0.78 ± 0.09	6.6·10^5^	2050	2050
5	0.36 ± 0.10	14.4·10^5^	6457	6457
6	0.33 ± 0.09	15.7·10^5^	6487	1297

^1^ Experiments were performed on a SA sensor chip with an IC_50_ of (3.25 ± 31) × 10^5^ nM for αMeMan.

## Supplementary Materials

The following are available online at www.mdpi.com/2073-4360/9/12/716/s1, Figure S1: ^1^H-NMR (600 MHz, D_2_O) of glycomacromolecule **1**, Figure S2: MALDI-TOF-MS spectrum of glycomacromolecule **1**, Figure S3: HR-MS (ESI^+^ Q-TOF) of glycomacromolecule **1**, Figure S4: ^1^H-NMR (600 MHz, D_2_O) of glycomacromolecule **2**, Figure S5: HPLC spectrum (95/5 H_2_O/MeCN with 0.1% formic acid (A) and 5/95 H_2_O/MeCN with 0.1% formic acid (B); 100% A to 50% A in 30 min) determined at 214 nm for glycomacromolecule **2** showing the thioether side product formation, Figure S6: MALDI spectrum for glycomacromolecule **2** showing the thioether side product formation, Figure S7: HR-MS (ESI^+^ Q-TOF) of glycomacromolecule **2**, Figure S8: ^1^H-NMR (600 MHz, D_2_O) of glycomacromolecule **3**, Figure S9: MALDI-TOF-MS spectrum of glycomacromolecule **3**, Figure S10: HR-MS (ESI^+^ Q-TOF) of glycomacromolecule **3**, Figure S11: ^1^H-NMR (300 MHz, D_2_O) of glycomacromolecule **1a**, Figure S12: MALDI-TOF-MS spectrum of glycomacromolecule **1a**, Figure S13: HR-MS (ESI^+^ Q-TOF) of glycomacromolecule **1a**, Figure S14: ^1^H-NMR (300 MHz, D_2_O) of glycomacromolecule **3a**, Figure S15: MALDI-TOF-MS spectrum of glycomacromolecule **3a**, Figure S16: HR-MS (ESI^+^ Q-TOF) of glycomacromolecule **3a**, Figure S17: TEM images of citrate stabilized gold nanoparticles with different magnifications. The TEM derived diameter is 14.1 ± 1.2 nm, Figure S18: TEM derived histogram of citrate stabilized gold nanoparticles**,** Figure S19: UV-Vis absorbance spectra of citrate stabilized AuNPs and glyco-AuNPs **4**–**6**, Figure S20: (a) Autocorrelation functions and (b) cumulant plot for citrate stabilized NPs and glyco-AuNPs **4**–**6** in water, Figure S21: Sodium chloride and LBB stability of citrate stabilized AuNPs and Glyco-AuNPs **4**–**6**: UV-Vis absorbance spectra of glyco Au-NPs **4** (a), **5** (b), **6** (c) and citrate stabilized AuNPs (d) with increasing sodium chloride concentration after 24 h; (e) normalized absorbance (normalized to absorbance at 400 nm) at 700 nm in dependence of sodium chloride concentration for citrate stabilized AuNPs and Glyco-AuNPs **4**–**6**; (f) UV-Vis spectra of glyco-AuNPs **4**–**6** in water and LBB after 24 h, Figure S22: UV-Vis absorbance spectra of glyco-AuNPs **4**–**6** at serial dilutions of Con A after addition of αMeMan in final concentration of 60 mM, Figure S23: UV-Vis absorbance spectra of glyco-AuNPs **4**–**6** at serial dilutions of BSA, Figure S24: Kinetic analysis of glyco-AuNP interaction with Con A by DLS measurements at *K*_D,app_ concentration of Con A for glyco-AuNP **4**–**6**. The average of three independent measurements is shown and the error bars correspond to the standard deviation in R_H_. The error bars of the time are in the range of the symbol size, Figure S25: Exemplary SPR sensorgram for solutions of 100 nM Con A incubated with serial dilutions of αMeMan and the resulting IC_50_ curve fitted to Hill1 function, Figure S26: Exemplary SPR sensorgram for solutions of 100 nM Con A incubated with three representative concentrations of d-(+)-galactose and the resulting relative response plotted against d-(+)-galactose concentration, Figure S27: Exemplary SPR sensorgram for solutions of 100 nM Con A incubated with serial dilutions of glyco-AuNP **4** and the resulting IC_50_ curve fitted to Hill1 function, Figure S28: Exemplary SPR sensorgram for solutions of 100 nM Con A incubated with serial dilutions of glyco-AuNP **5** and the resulting IC_50_ curve fitted to Hill1 function, Figure S29: Exemplary SPR sensorgram for solutions of 100 nM Con A incubated with serial dilutions of *N*-ethylmaleimide capped glycomacromolecule **3a** and the resulting IC_50_ curve fitted to Hill1 function, Figure S30: Exemplary SPR sensorgram for solutions of 100 nM Con A incubated with serial dilutions of *N*-ethylmaleimide capped glycomacromolecule **1a** and the resulting IC_50_ curve fitted to Hill1 function.
