# Correction: Sophia, B.; et al. Presenting Precision Glycomacromolecules on Gold Nanoparticles for Increased Lectin Binding. *Polymers* 2017, *9*, 716

**DOI:** 10.3390/polym11091426

**Published:** 2019-08-30

**Authors:** Sophia Boden, Kristina G. Wagner, Matthias Karg, Laura Hartmann

**Affiliations:** 1 Institute of Organic Chemistry and Macromolecular Chemistry, Heinrich-Heine-University Düsseldorf, Universitätsstraße 1, 40225 Düsseldorf, Germany; 2 Institute of Physical Chemistry, Heinrich-Heine-University Düsseldorf, Universitätsstraße 1, 40225 Düsseldorf, Germany

In the original version of this Article [1], an error occurred in the “Results and Discussion” section, under the subheading “Preparation and Characterization of Glyco-AuNPs”. The section contains a calculation error in Table 1. The calculation for the degree of glycomacromolecule functionalization per NP (TBO) is wrong due to the fact that one of the dilution steps in the TBO procedure was omitted in the calculation. This is why all given values for this assay are too small by a constant factor of 6.67. However, based on previously reported surface loading values for comparable systems in the literature [86,87], we believe that the values determined by the phenol sulphuric acid method (PSA) are more reliable. Therefore, the average degree of functionalization for the following sections only slightly changes within the error margins and does not affect the conclusions of the article. Nevertheless, for consistency, values normalized to the degree of functionalization were calculated now using numbers from the PSA assay. In Table 2, K_D,app_ per ligand and per Mannose changes for compounds 4 and 5 (4: from 67 to 65, 5: from 45 to 47). In Table 4, RIP per ligand and per Mannose changes for compounds **4** and **5** (**4**: from 2050 to 2115, **5**: from 6457 to 6234). The authors apologize for the error. 

Corrected Table 1:

We added to the manuscript: […] However, this method is highly pH sensitive and as such could lead to unreliable results as glyco-AuNPs were dispersed in MilliQ water affecting the final pH value. Based on previously reported surface loading values for comparable systems in the literature, we believe that the values determined by PSA are more reliable for this system [86,87]. 

We removed the sentence: The determined degree of glycomacromolecule functionalization for both methods is in good agreement (Table 1).

New references (numbering was adapted accordingly in the corrected manuscript):

86. Rahme, K.; Chen, L.; Hobbs, R.G.; Morris, M.A.; O’Driscoll, C.; Holmes, J.D. Correction: PEGylated gold nanoparticles: polymer quantification as a function of PEG lengths and nanoparticle dimensions. *RSC Adv.* **2017**, *7*, 8798–8799, doi:10.1039/c7ra90006f.
87. Kennedy D.C.; Orts-Gil, G.; Lai, C.-H.; Müller, L.; Haase, A.; Luch, A.; Seeberger, P.H. Carbohydrate functionalization of silver nanoparticles modulates cytotoxicity and cellular uptake. *J. Nanobiotechnol.* **2014**, *12*, 59, doi:10.1186/s12951-014-0059-z.

## Figures and Tables

**Table 1:** The DLS derived hydrodynamic radii (R_H_) and degree of glycomacromolecule functionalization determined by phenol sulphuric acid method (PSA) and Toluidine Blue O-Titration (TBO) and derived average degree of carbohydrate functionalization and occupied surface area.

AuNP	R_H_ in Water (nm)	Degree of Glycomacromolecule Functionalization per NP (PSA)	Degree of Glycomacromolecule Functionalization per NP (TBO)	Average Degree of Carbohydrate Functionalization per NP (PSA)	Average Occupied Surface Area per Glycomacromolecule (PSA) (nm^2^)
Citrate stabilized	8.5 ± 0.1	-	-	-	-
4	11.7 ± 0.1	312 ± 218	2210 ± 553	~312	~2.0
5	13.3 ± 0.3	231 ± 98	1430 ± 377	~231	~2.7
6	13.5 ± 0.1	242 ± 2	1620 ± 206	~1210	~2.5
