# Supplementary material for: Presenting Precision Glycomacromolecules on Gold Nanoparticles for Increased Lectin Binding
Source: Polymers (Basel). 2017 Dec 14;9(12):716. doi: 10.3390/polym9120716 (PMC6418785; doi:10.3390/polym9120716)
Supplement: Supplementary file 1 [file polymers-09-00716-s001.pdf]

Supporting Information

Presenting Precision Glycomacromolecules on Gold Nanoparticles for Increased Lectin Binding

Sophia Boden [1], Kristina G. Wagner [2], Matthias Karg [2] and Laura Hartmann [1,*]

[1] Institute of Organic Chemistry and Macromolecular Chemistry, Heinrich-Heine-University Düsseldorf, Universitätsstraße 1, 40225 Düsseldorf, Germany; Laura.Hartmann@hhu.de
[2] Institute of Physical Chemistry, Heinrich-Heine-University Düsseldorf, Universitätsstraße 1, 40225 Düsseldorf, Germany; Matthias.Karg@hhu.de
* Correspondence: Laura.Hartmann@hhu.de; Tel.: +49-211-81-10360

1. Synthesis of Thiol Functionalized Precision Glycomacromolecules

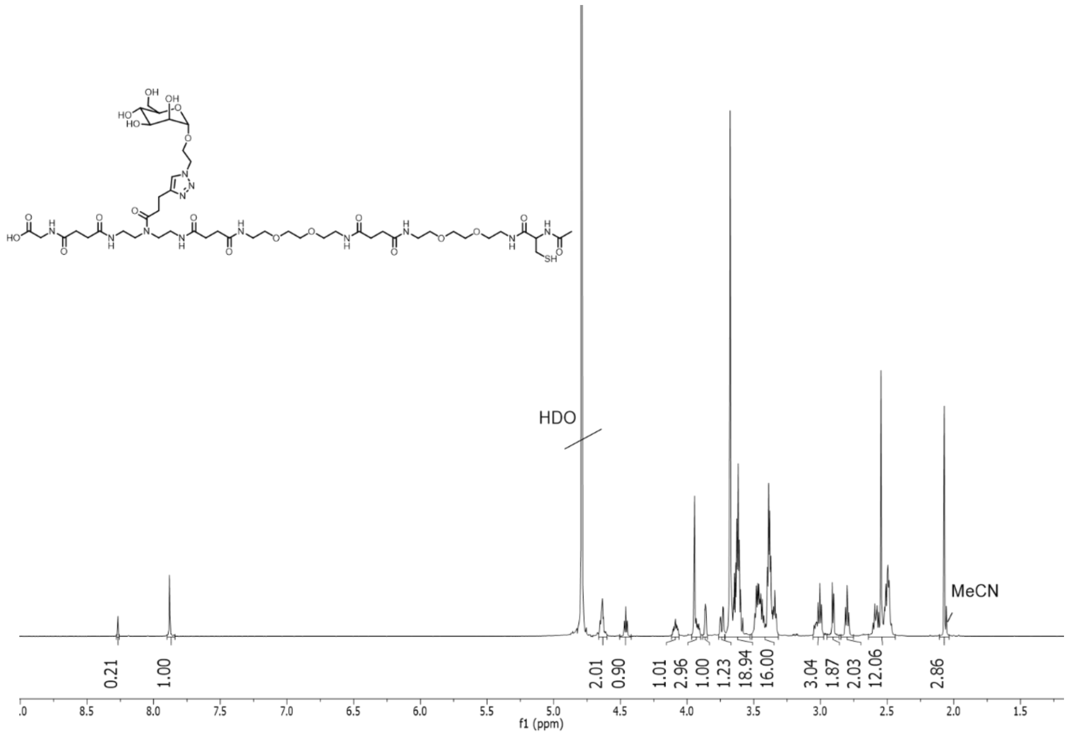

Figure S1. ^{1}H-NMR (600 MHz, D_2O) of glycomacromolecule 1.

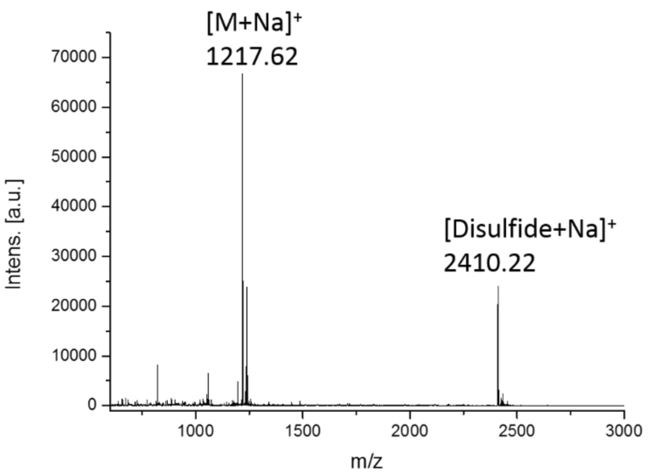

Figure S2. MALDI-TOF-MS spectrum of glycomacromolecule 1.

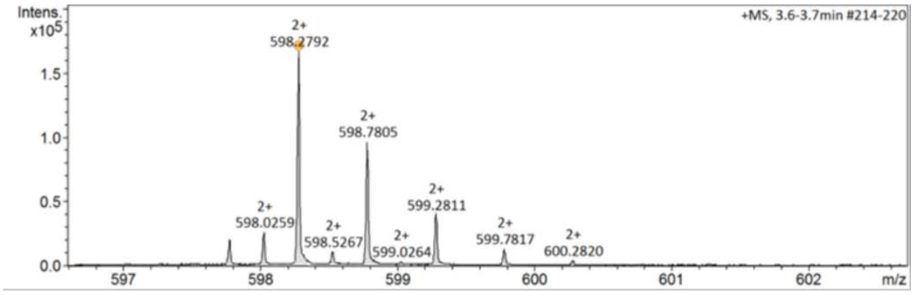

Figure S3. HR-MS (ESI^+ Q-TOF) of glycomacromolecule 1.

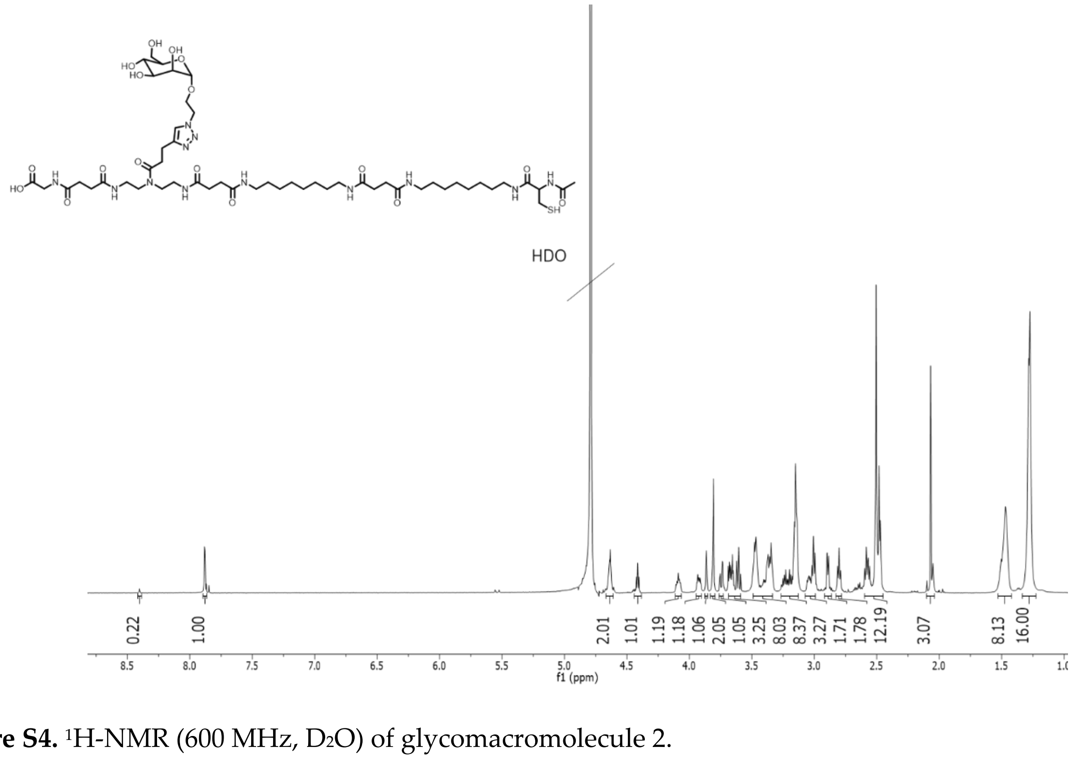

Figure S4. ^{1}H-NMR (600 MHz, D_2O) of glycomacromolecule 2.

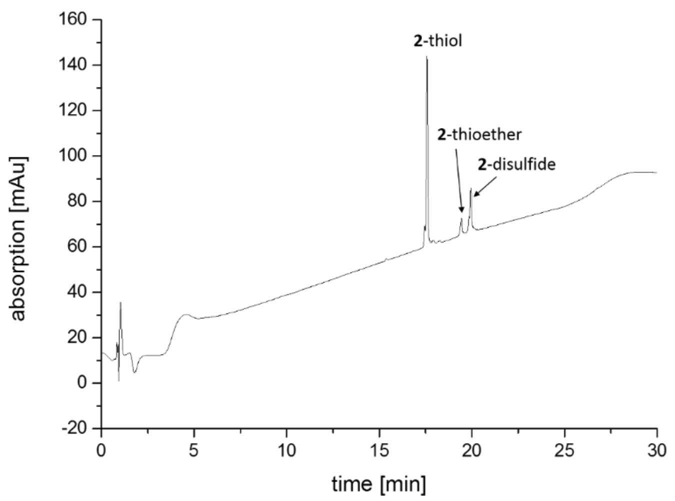

Figure S5. HPLC spectrum (95/5 H_2O/MeCN with 0.1% formic acid (A) and 5/95 H_2O/MeCN with 0.1% formic acid (B); 100% A to 50% A in 30min) determined at 214 nm of glycomacromolecule 2 showing the thioether side product formation.

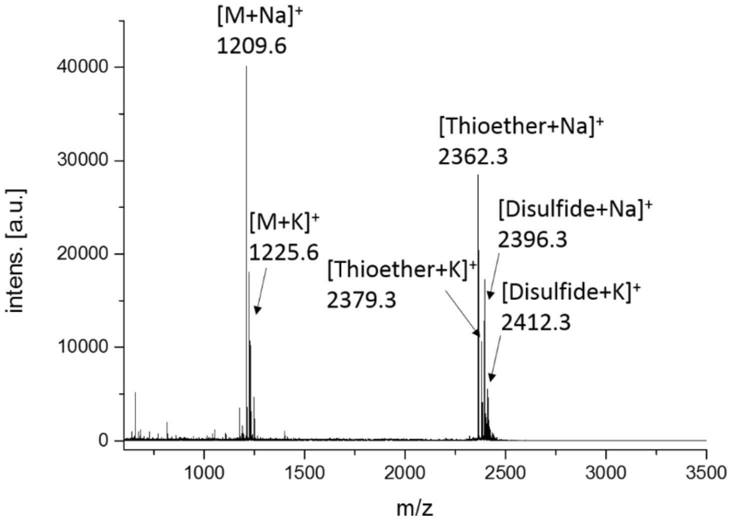

Figure S6. MALDI-TOF-MS spectrum of glycomacromolecule 2 showing the thioether side product formation.

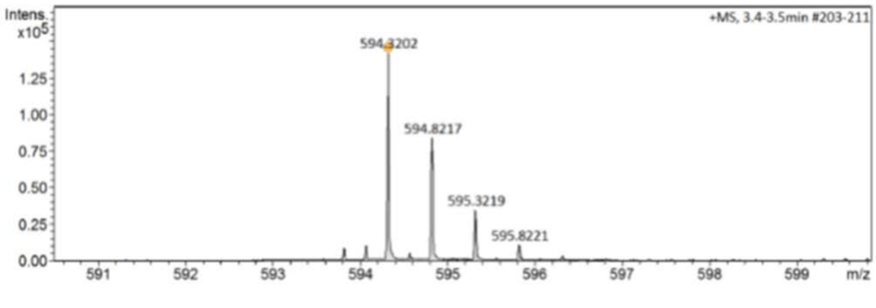

Figure S7. HR-MS (ESI^+ Q-TOF) of glycomacromolecule 2.

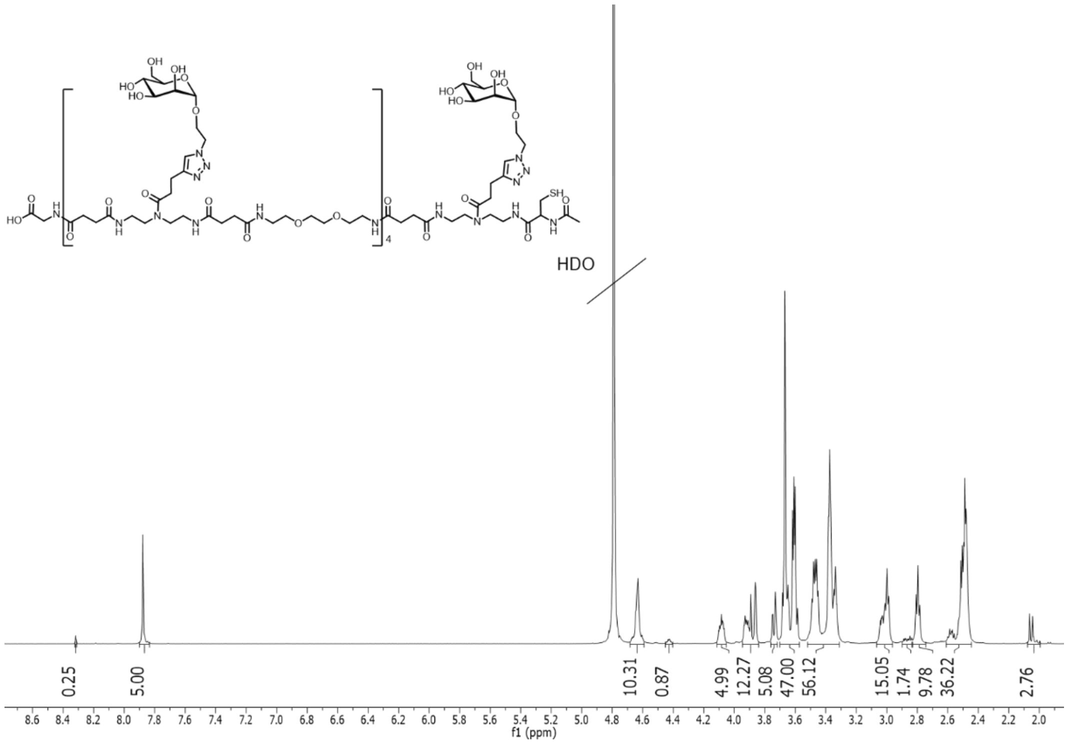

Figure S8. ^{1}H-NMR (600 MHz, D_2O) of glycomacromolecule 3.

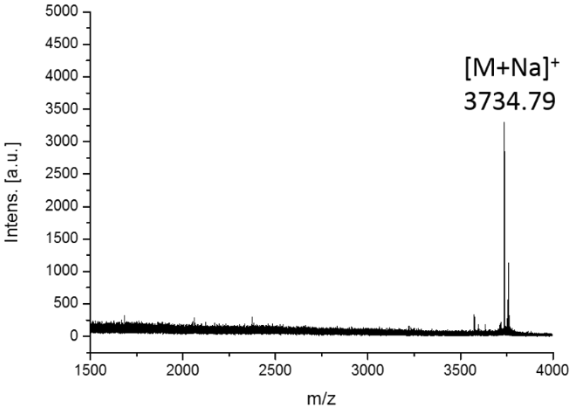

Figure S9. MALDI-TOF-MS spectrum of glycomacromolecule 3.

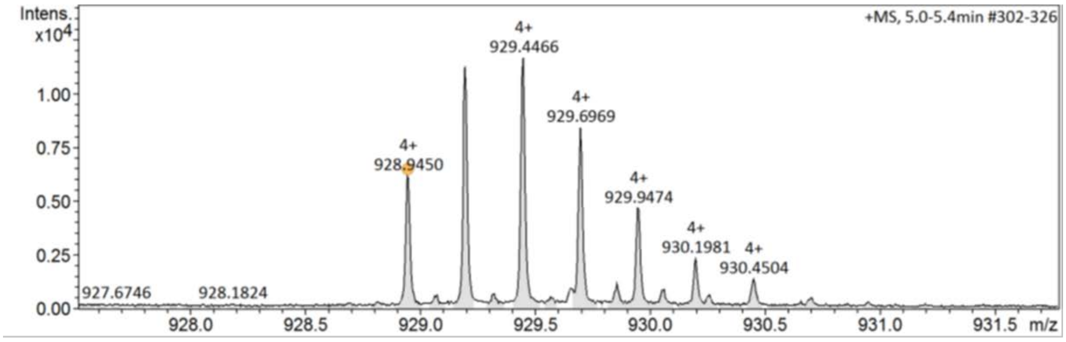

Figure S10. HR-MS (ESI^{+} Q-TOF) of glycomacromolecule 3.

2. Synthesis of *N*-Ethylmaleimide capped Precision Glycomacromolecules

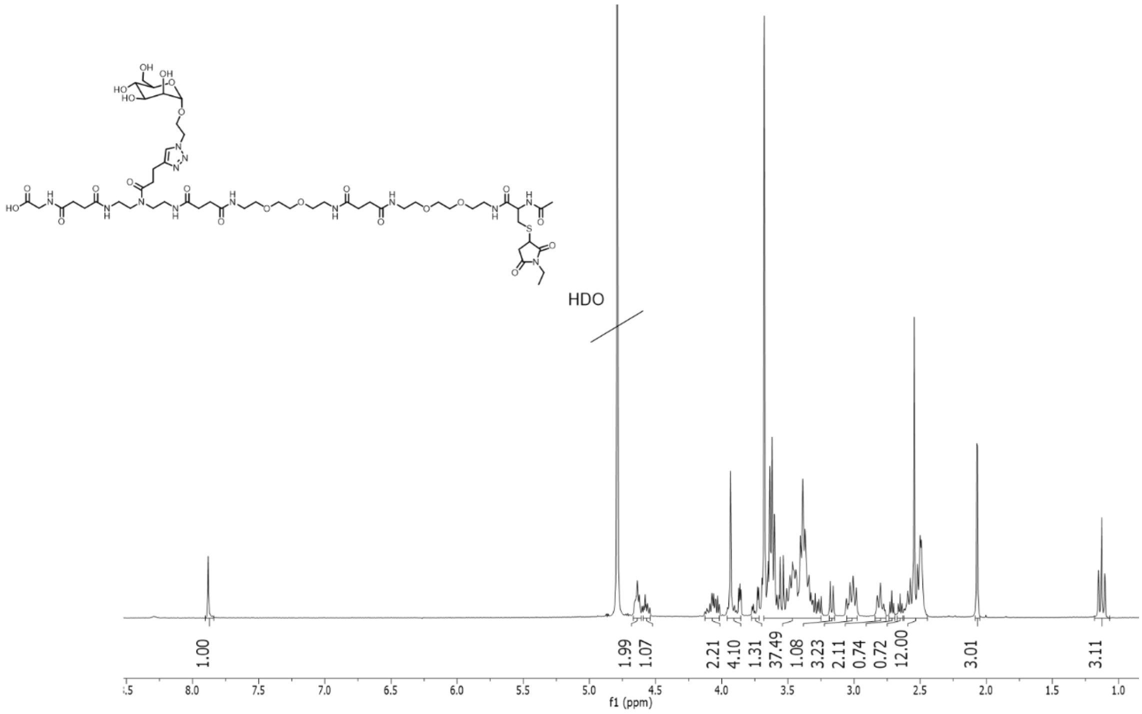

Figure S11. ^{1}H-NMR (300 MHz, D_2O) of glycomacromolecule 1a.

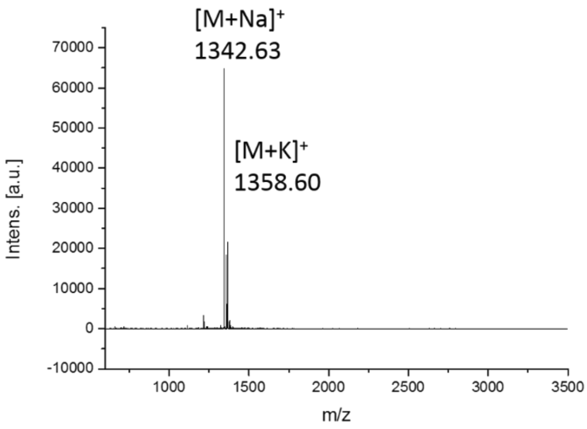

Figure S12. MALDI-TOF-MS spectrum of glycomacromolecule 1a.

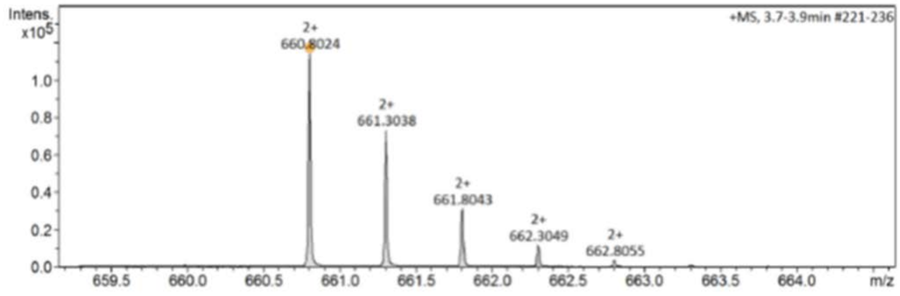

Figure S13. HR-MS (ESI$^+$ Q-TOF) of glycomacromolecule 1a.

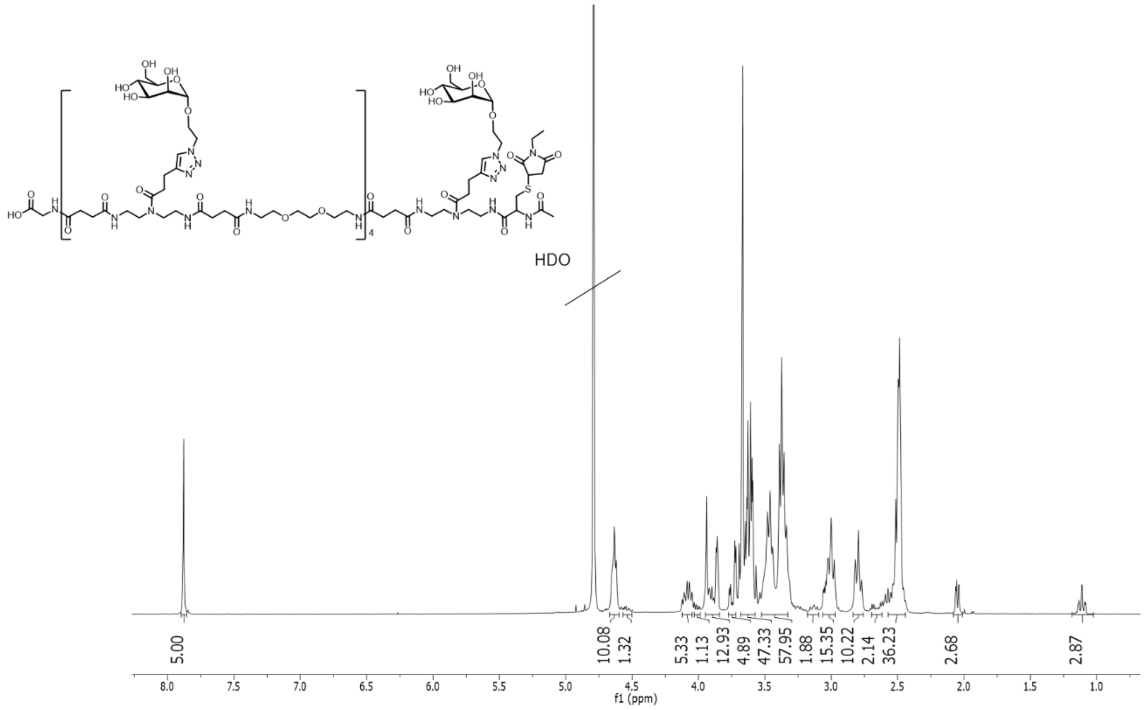

Figure S14. ^{1}H-NMR (300 MHz, D_2O) of glycomacromolecule 3a.

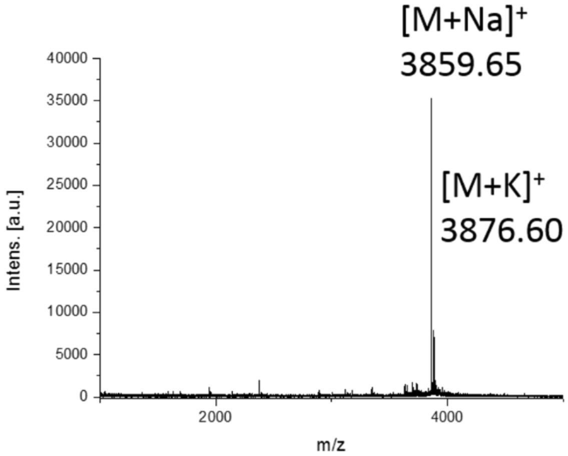

Figure S15. MALDI-TOF-MS spectrum of glycomacromolecule 3a.

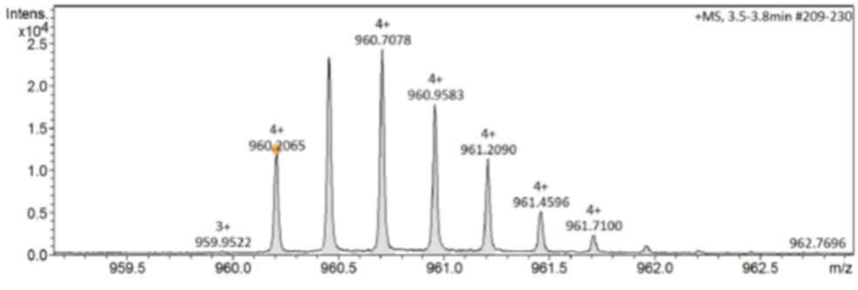

Figure S16. HR-MS (ESI^{+} Q-TOF) of glycomacromolecule 3a.

3. Characterization of Citrate Stabilized NPs and Glyco-AuNPs

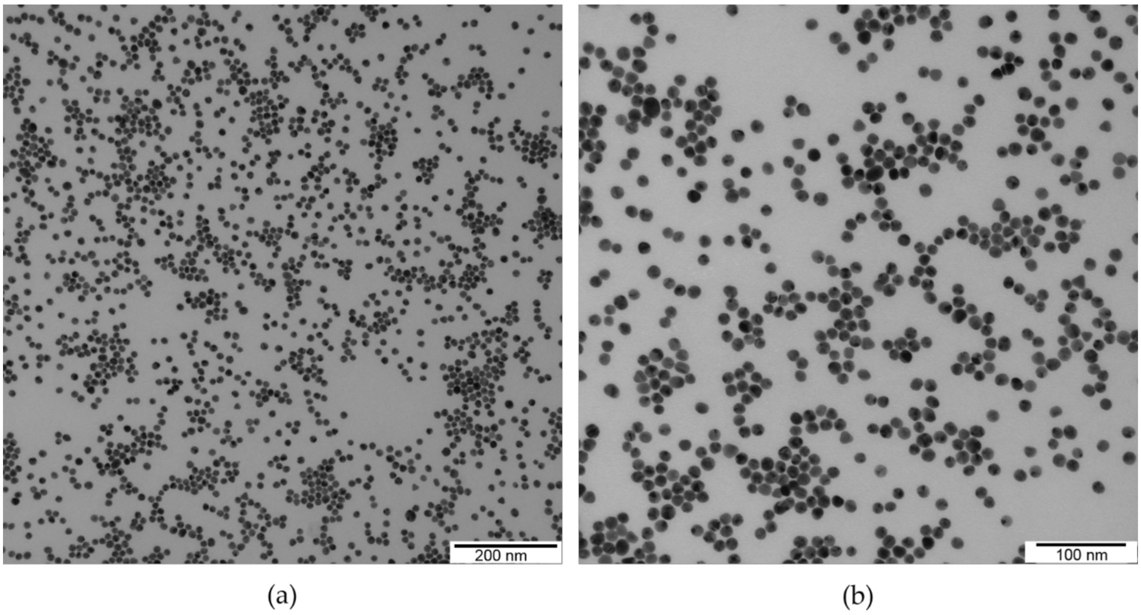

Figure S17. TEM images of citrate stabilized gold nanoparticles with different magnifications. The TEM derived diameter is 14.1±1.2 nm.

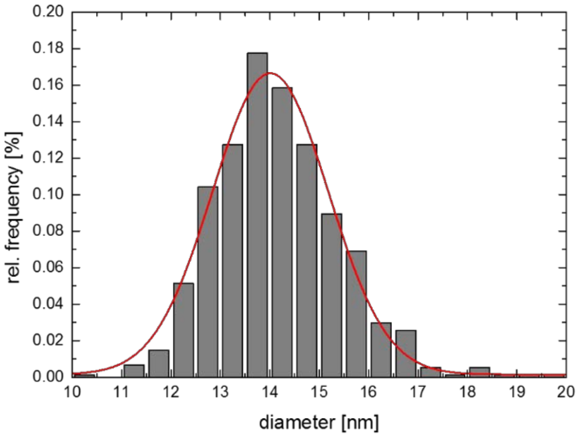

Figure S18. TEM derived histogram of citrate stabilized gold nanoparticles.

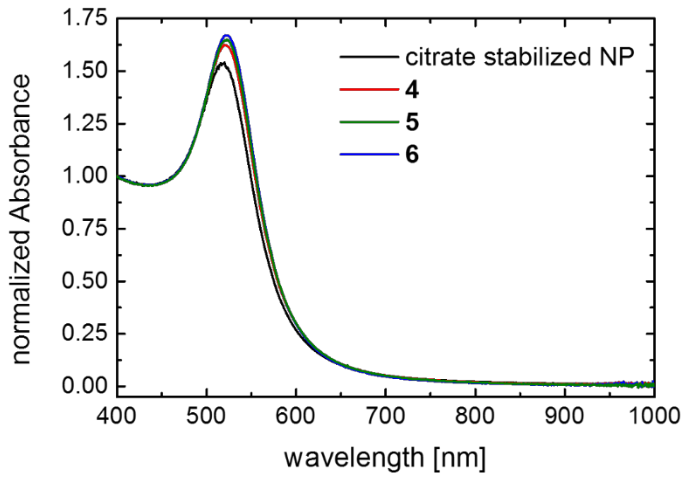

Figure S19. UV-Vis absorbance spectra of citrate stabilized AuNPs and glyco-AuNPs **4**-**6**.

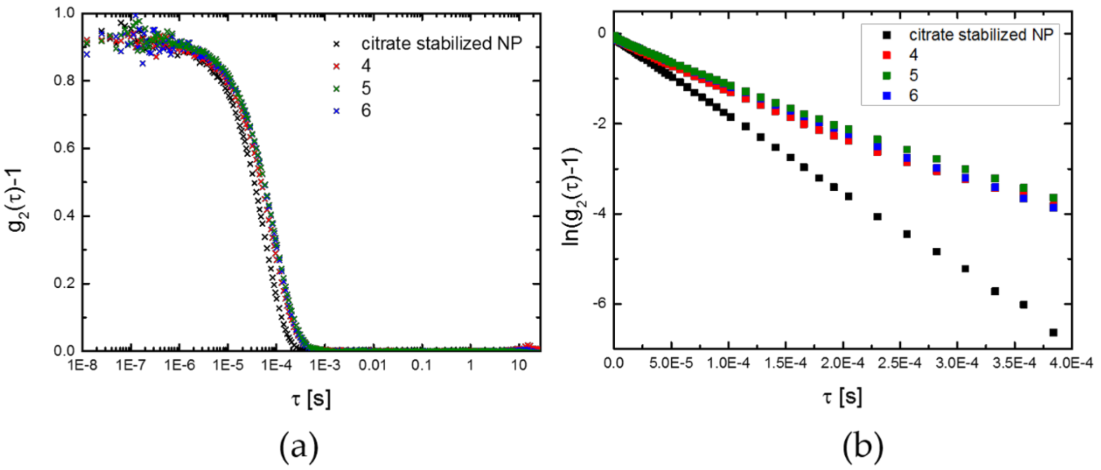

Figure S20. (a) Autocorrelation functions and (b) cumulant plot for citrate stabilized NPs and glyco-AuNPs **4**-**6** in water.

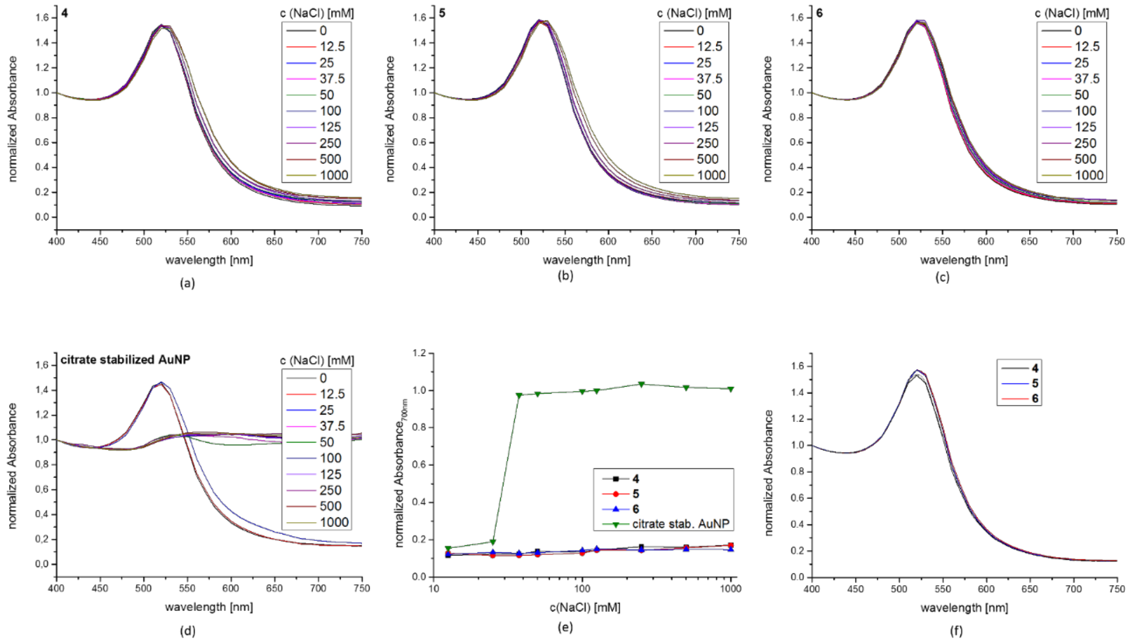

Figure S21. Sodium chloride and LBB stability of citrate stabilized AuNPs and Glyco-AuNPs **4**-**6**: UV-Vis absorbance spectra of glyco Au-NPs **4** (a), **5** (b), **6** (c) and citrate stabilized AuNPs (d) with increasing sodium chloride concentration after 24 h; (e) normalized absorbance (normalized to absorbance at 400 nm) at 700 nm in dependence of sodium chloride concentration for citrate stabilized AuNPs and Glyco-AuNPs **4**-**6**; (f) UV-Vis spectra of glyco-AuNPs **4**-**6** in water and LBB after 24 h.

4. Binding Studies with the Model Lectin Concanavalin A (Con A)

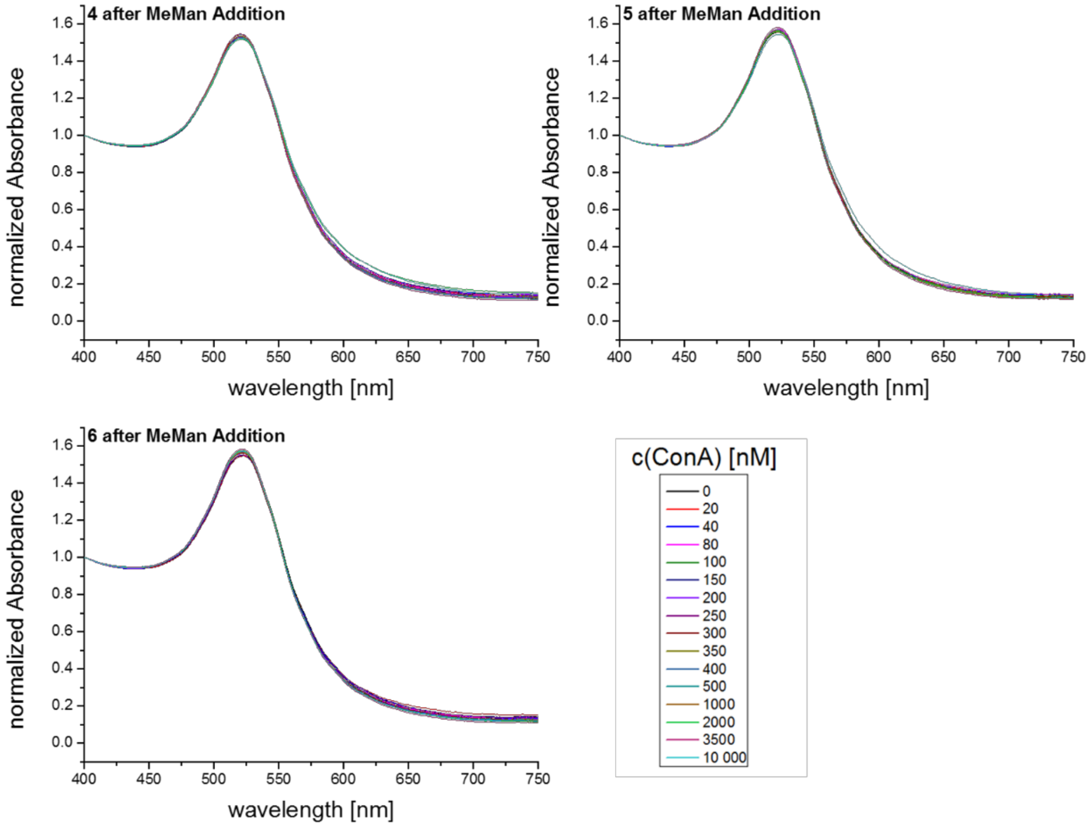

Figure S22. UV-Vis absorbance spectra of glyco-AuNPs **4-6** at serial dilutions of Con A after addition of αMeMan in final concentration of 60 mM.

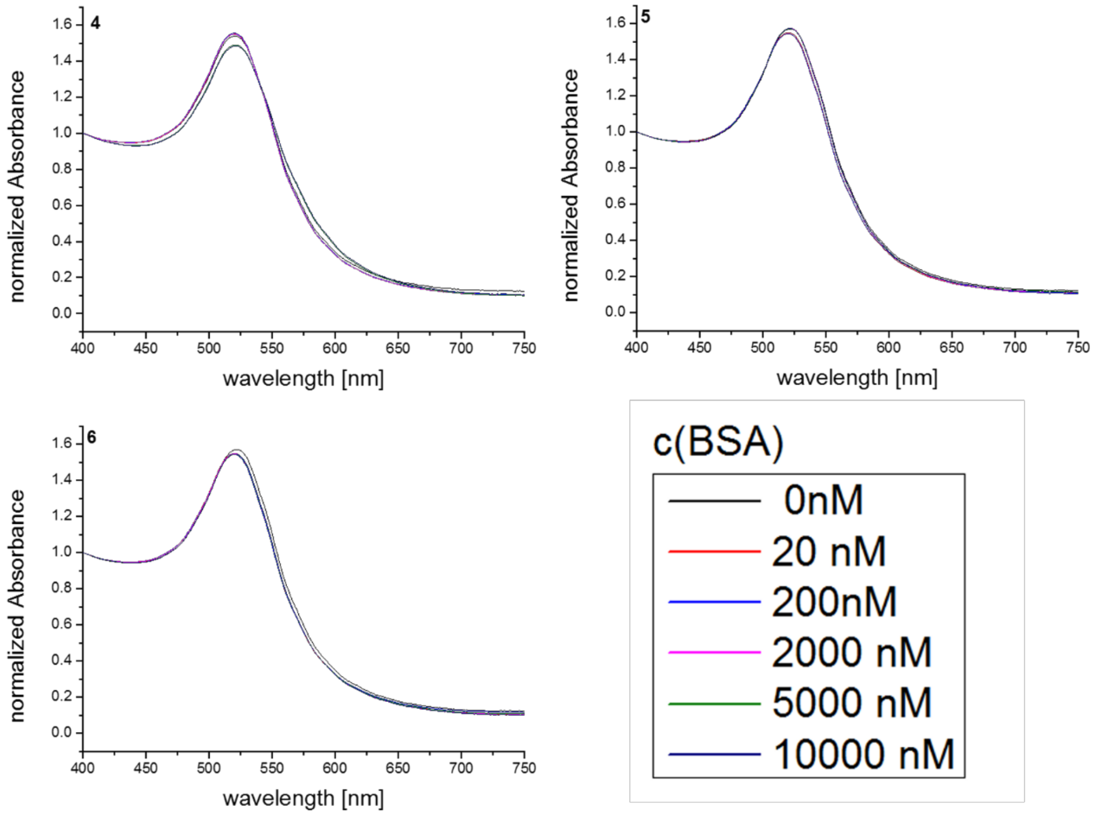

Figure S23. UV-Vis absorbance spectra of glyco-AuNPs **4**-**6** at serial dilutions of BSA.

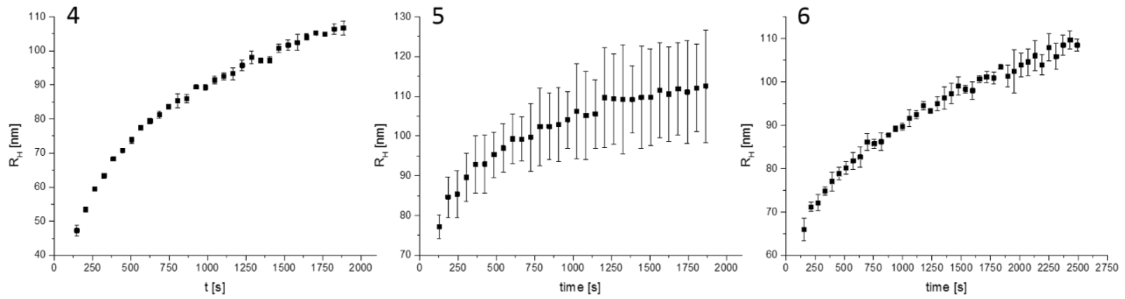

Figure S24. Kinetic analysis of glyco-AuNP interaction with Con A by DLS measurements at $K_{D,app}$ concentration of Con A for glyco-AuNP **4**, **5** and **6**. The average of three independent measurements is shown and the error bars correspond to the standard deviation in R_H. The error bars of the time are in the range of the symbol size.

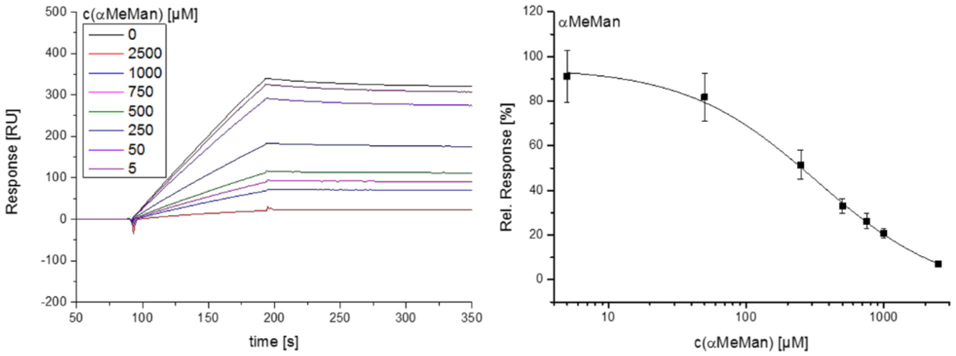

Figure S25. Exemplary SPR sensorgram for solutions of 100 nM Con A incubated with serial dilutions of αMeMan and the resulting IC_{50} curve fitted to Hill1 function.

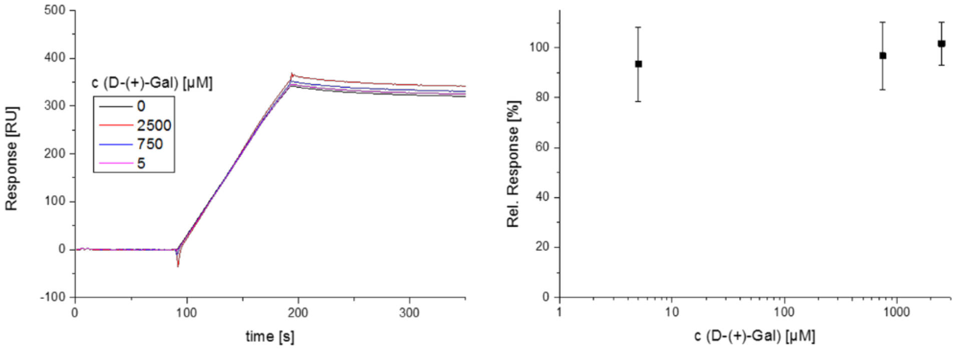

Figure S26. Exemplary SPR sensorgram for solutions of 100 nM Con A incubated with three representative concentrations of D-(+)-galactose and the resulting relative response plotted against D-(+)-galactose concentration.

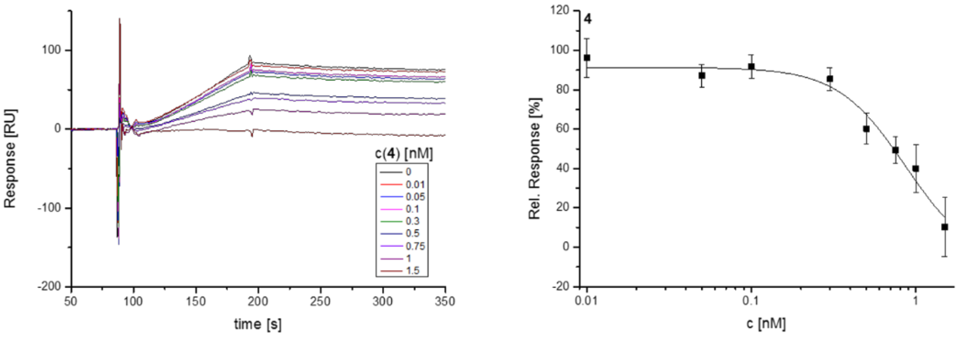

Figure S27. Exemplary SPR sensorgram for solutions of 100 nM Con A incubated with serial dilutions of glyco-AuNP **4** and the resulting IC_{50} curve fitted to Hill1 function.

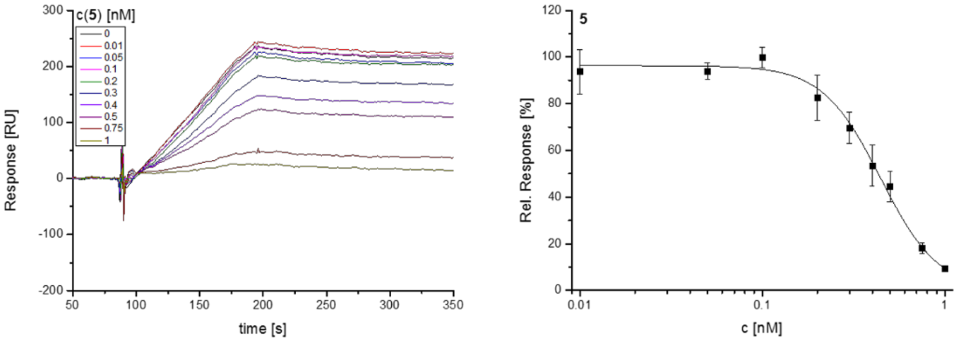

Figure S28. Exemplary SPR sensorgram for solutions of 100 nM Con A incubated with serial dilutions of glyco-AuNP **5** and the resulting IC_{50} curve fitted to Hill1 function.

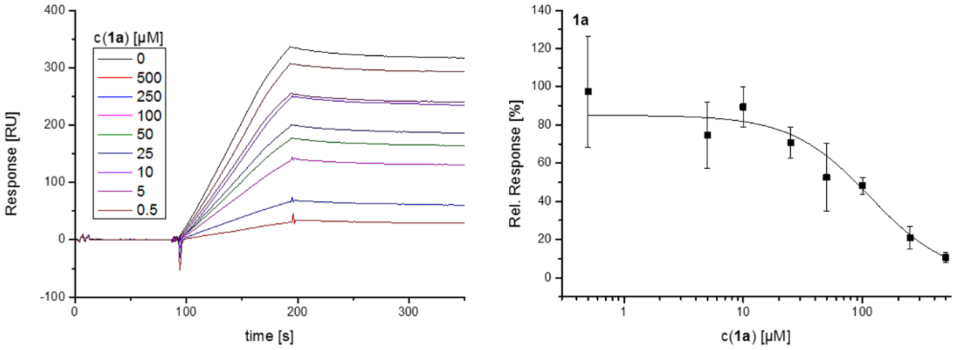

Figure S29. Exemplary SPR sensorgram for solutions of 100 nM Con A incubated with serial dilutions of *N*-ethylmaleimide capped glycomacromolecule **1a** and the resulting IC_{50} curve fitted to Hill1 function.

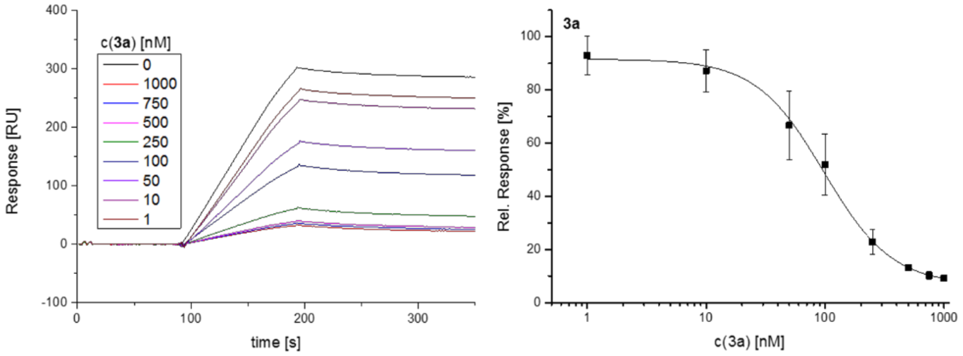

Figure S30. Exemplary SPR sensorgram for solutions of 100 nM Con A incubated with serial dilutions of *N*-ethylmaleimide capped glycomacromolecule **3a** and the resulting IC_{50} curve fitted to Hill1 function.
